# Terpenoids From the Coral-Derived Fungus *Trichoderma harzianum* (XS-20090075) Induced by Chemical Epigenetic Manipulation

**DOI:** 10.3389/fmicb.2020.00572

**Published:** 2020-04-03

**Authors:** Ting Shi, Chang-Lun Shao, Yang Liu, Dong-Lin Zhao, Fei Cao, Xiu-Mei Fu, Jia-Yin Yu, Jing-Shuai Wu, Zhen-Kun Zhang, Chang-Yun Wang

**Affiliations:** ^1^Key Laboratory of Marine Drugs, the Ministry of Education of China, School of Medicine and Pharmacy, Ocean University of China, Qingdao, China; ^2^Laboratory for Marine Drugs and Bioproducts, Qingdao National Laboratory for Marine Science and Technology, Qingdao, China; ^3^Institute for Insect Biotechnology, Justus-Liebig-University of Giessen, Giessen, Germany; ^4^Department of Bioresources, Fraunhofer Institute for Molecular Biology and Applied Ecology, Giessen, Germany; ^5^Marine Agriculture Research Center, Tobacco Research Institute of Chinese Academy of Agricultural Sciences, Qingdao, China; ^6^Key Laboratory of Pharmaceutical Quality Control of Hebei Province, College of Pharmaceutical Sciences, Hebei University, Baoding, China; ^7^Institute of Evolution and Marine Biodiversity, Ocean University of China, Qingdao, China

**Keywords:** Coral-derived fungus, *Trichoderma harzianum*, Chemical epigenetic manipulation, Diterpenoids, Sesquiterpenoids

## Abstract

The soft coral-derived fungus *Trichoderma harzianum* (XS-20090075) was found to be a potential strain to produce substantial new compounds in our previous study. In order to explore its potential to produce more metabolites, chemical epigenetic manipulation was used on this fungus to wake its sleeping genes, leading to the significant changes of its secondary metabolites by using a histone deacetylase (HDAC) inhibitor. The most obvious difference was the original main products harziane diterpenoids were changed into cyclonerane sesquiterpenoids. Three new terpenoids were isolated from the fungal culture treated with 10 μM sodium butyrate, including cleistanthane diterpenoid, harzianolic acid A (**1**), harziane diterpenoid, harzianone E (**2**), and cyclonerane sesquiterpenoid, 3,7,11-trihydroxy-cycloneran (**3**), together with 11 known sesquiterpenoids (**4**–**14**). The absolute configurations of **1**–**3** were determined by single-crystal X-ray diffraction, ECD and OR calculations, and biogenetic considerations. This was the first time to obtain cleistanthane diterpenoid and africane sesquiterpenoid from genus *Trichoderma*, and this was the first chlorinated cleistanthane diterpenoid. These results demonstrated that the chemical epigenetic manipulation should be an efficient technique for the discovery of new secondary metabolites from marine-derived fungi.

## Graphical Abstract

Terpenoids from *T. harzianum* induced by chemical epigenetic manipulation.

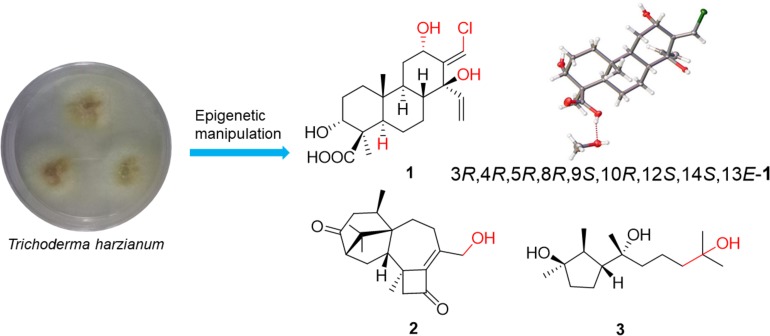

## Introduction

Marine fungi have been proved to possess the potential ability to produce structurally unique and biologically active secondary metabolites (Blunt et al., 2018). However, it has become a crucial issue to discover microbial natural products due to repeating isolation of known compounds at the traditional methods involving bulk culture of the organism and subsequent fractionation and bioassay to determine if specific fractions hold any bioactive metabolites (Spraker and Keller, 2014). It has been revealed that fungi possess far more gene clusters encoding secondary metabolites than

their characterized compounds (Khaldi et al., 2010). In order to solve this challenge, a number of manipulations have been used to regulate the production of secondary metabolites from fungi, such as One strain many compounds (OSMAC) (Pan et al., 2019), co-culture (Yu et al., 2019), interspecies crosstalk (Wang et al., 2019), and heterologous expression (Huo et al., 2019; Zhang et al., 2019). Among these methods, chemical epigenetic manipulation has been demonstrated to be a promising strategy to wake the silent biosynthetic gene clusters to obtain novel compounds and has been applied to the marine fungi (Asai et al., 2013). For instance, histone deacetylase (HDAC) inhibitor, suberoylanilide hydroxamic acid (SAHA), was applied to an algicolous strain of *Aspergillus wentii*, giving rise to three new norditerpenoids with potent bioactivities (Li et al., 2017). Similarly, treating deepsea-derived *Eutypella* sp. fungus with a combination of HDAC inhibitor (SAHA) and DNA methyltransferase (DNMT) inhibitor (5-azacytidine) resulted in the discovery of three new eremophilane-type sesquiterpenoids with nitric oxide inhibitory activities (Niu et al., 2018). These cases might demonstrate that chemical epigenetic manipulation could efficiently excavate novel secondary metabolites from marine-derived fungi. However, the successful examples of chemical epigenetic manipulation applied to marine-derived fungi are not abundant enough to confirm the conclusion.

*Trichoderma* species are widespread, highly competitive soil-borne fungi. They display a successful antagonism against a variety of other fungi (Hanson, 2005). Fungus *Trichoderma harzianum* is known to be a biocontrol agent against phytopathogenic fungi extensively applied in agriculture (Hassan et al., 2019; Dufresne et al., 2020). This fungus has some applications in other aspects of agriculture, such as improving drought tolerance in rice genotypes (Pandey et al., 2016) and increasing plant productivity (Poveda et al., 2019). One of the mechanism of these bioactivities of *T. harzianum* is considered to be its ability to produce metabolites with various activities, such as antifungal harzianopyridone (Ahluwalia et al., 2015) and 6-pentyl-α-pyrone (Chen et al., 2012), plant growth promoter harzianic acid (Vinale et al., 2013), and plant growth regulator harzianolide (Cai et al., 2013). Marine-derived *T. harzianum* can also produce substantial active secondary metabolites (Liang et al., 2019; Yamada et al., 2019; Zhao et al., 2019). More than 60 compounds have been isolated from marine-derived *T. harzianum* that further demonstrated the potential ability of this fungus to produce natural products with diverse structures. Up to now, there has no research to study the secondary metabolites of *T. harzianum* through epigenetic modification.

During our ongoing investigation to discover bioactive marine natural products, we have also obtained new metabolites from marine-derived fungi by using chemical epigenetic modification (Zhang et al., 2014; Chen et al., 2016; Wu et al., 2019, 2020). In our previous work, a series of harziane diterpenoids and hydroxyanthraquinones have been discovered from the fungus *T. harzianum* (XS-20090075) isolated from a soft coral collected from the South China Sea (Shi et al., 2018; Zhao et al., 2019). In order to obtain more new bioactive compounds, chemical epigenetic manipulation was employed on this fungal strain to mine its potential ability to produce metabolic products. Screening chemical epigenetic modifying agents resulted in the significant changes of its metabolic profile by using HDAC inhibitors. Subsequently, besides harziane diterpenoid, new metabolic products were discovered from this strain including cleistanthane diterpenoids and cyclonerane sesquiterpenoids. Herein, we report the epigenetic modification on this fungus, and the isolation, structural characterization and bioactivity evaluation of these metabolites.

## Materials and Methods

### General Experimental Procedures

Optical rotations were measured on a JASCO P-1020 digital polarimeter. UV spectra were recorded on a Beckman DU 640 spectrophotometer. ECD spectra were obtained on a Jasco J-815-150S circular dichroism spectrometer. IR spectra were recorded on a Nicolet-Nexus-470 spectrometer using KBr pellets. NMR spectra were measured on an Agilent DD2 500 MHz NMR spectrometer (500 MHz for ^1^H and 125 MHz for ^13^C), using TMS as an internal standard. The ESIMS and HRESIMS spectra were obtained from a Micromass Q-TOF spectrometer and a Thermo Scientific LTQ Orbitrap XL spectrometer, respectively. The crystallographic data were collected on a Bruker APEX-II CCD diffractometer equipped with graphite monochromatized Cu Kα radiation. Semi-preparative HPLC was performed on a Waters 1525 system coupled with a Waters 2996 photodiode array detector. A Kromasil C_18_ semi-preparative HPLC column (250 × 10 mm, 5 μm) was used. Silica gel (Qing Dao Hai Yang Chemical Group Co.; 200–300 mesh), Sephadex LH-20 (Amersham Biosciences) and octadecylsilyl silica gel (Unicorn; 45–60 μm) were used for column chromatography (CC). Precoated silica gel GF_254_ plates (Yantai Zifu Chemical Group Co., Yantai, China) were used for thin-layer chromatography.

### Fungal Material

The fungal strain *T. harzianum* (XS-20090075) was isolated from a piece of fresh tissue from the inner part of an unidentified soft coral, collected from Xisha Islands coral reef in the South China Sea in September 2009. The strain was deposited in the Key Laboratory of Marine Drugs, the Ministry of Education of China, School of Medicine and Pharmacy, Ocean University of China, Qingdao, China, with the GenBank (NCBI) accession number KU866299.

### Extraction and Isolation

The fungal strain *T. harzianum* (XS-20090075) was fermented in a rice medium treated with 10 μM sodium butyrate in 1000 mL Erlenmeyer flasks (100 g rice, 150 mL water, 0.165 mg sodium butyrate and 4.50 g natural sea salt (Qingdao Salt Franchise Co., Ltd.) in each flask, 100 flasks) at room temperature for 45 days. The difference with the previous cultured 28 days (Zhao et al., 2019) was because that, compared with the 45 days of culture without epigenetic modifier, more different peaks of secondary metabolites appeared in HPLC profile of EtOAc extract. The fermented substrate was extracted with EtOAc (200 mL × 3 for each flask) and CH_3_OH/CH_2_Cl_2_ (200 mL × 3 for each flask), and the solvent was combined and concentrated *in vacuo* to afford a residue (15.0 g). The residue (15.0 g) was subjected to vacuum liquid chromatography (VLC) on silica gel using step gradient elution with EtOAc in petroleum ether (PE) (0-100%) and then with MeOH in EtOAc (0-100%) to afford three fractions (Fr.1-Fr.3). Fr.1 was isolated by CC on silica gel eluted with EtOAc in PE (0-100%) to afford four subfractions (Fr.1.1-Fr.1.4). Fr.1.3 was subjected to Sephadex LH-20 CC with CH_2_Cl_2_-CH_3_OH (v/v, 1:1), and then purified by repeated CC on silica gel gradient eluted with gradient EtOAc in PE to obtain **13** (2.7 mg) and **14** (2.7 mg). Fr.3 was isolated by gradient elution of ODS CC with MeOH in H_2_O (10-100%) to get 10 subfractions. Fr.3.7 was first subjected to Sephadex LH-20 CC with CH_2_Cl_2_-CH_3_OH (v/v, 1:1), then isolated on silica gel eluted with gradient EtOAc in PE (20-100%), and further purified on semi-preparative HPLC using an ODS column (Kromasil C18, 250 × 10 mm, 5 μm, 2 mL/min) eluted with 50% MeOH-H_2_O to get **4** (1.8 mg) and **8** (1.5 mg). Fr.3.8 was subjected to Sephadex LH-20 CC with CH_2_Cl_2_-CH_3_OH (v/v, 1:1) to get two subfractions. Fr.3.8.1 was isolated on repeated silica gel CC gradient eluted with PE-EtOAc to yield **2** (4.3 mg), **10** (1.8 mg), **11** (1.6 mg) and **12** (5.0 mg); Fr.3.8.2 was purified on HPLC (60% MeOH-H_2_O) to offer **3** (3.2 mg) and **9** (2.1 mg). Fr.3.9 was isolated on Sephadex LH-20 CC with CH_2_Cl_2_-CH_3_OH (v/v, 1:1) to provide two subfractions. Fr.3.9.1 was subjected on repeated silica gel CC gradient eluted with EtOAc in PE to afford **5** (9.6 mg) and **6** (6.3 mg); Fr.3.9.2 was purified on HPLC (60% MeOH-H_2_O) for **1** (2.0 mg). Fr.3.10 was purified on HPLC eluted with 40% MeOH-H_2_O to give **7** (1.1 mg).

Harzianolic acid (**1**): Yellow needle crystal; [α]${}_{\text{D}}^{22}$ + 26.3 (*c* 1.00, MeOH); UV (MeOH) λ_max_ (log ε) 250 (3.47), 280 (3.29) nm; IR (KBr) ν_max_ 3748, 3445, 2921, 1700, 1650, 1540, 1158, 560 cm^–1^; ^1^H and ^13^C NMR data, see Table 1 and Supplementary Figures S2–S12; ESIMS *m/z* 383.2 [M − H]^–^, isotopic peak 385.2 [M − H]^–^, exhibited chlorine isotope peak ratio of 3:1; HRESIMS *m/z* 383.1631 [M − H]^–^ (calcd. for C_20_H_28_O_5_^35^Cl, 383.1620), 385.1604 [M − H]^–^ (calcd. for C_20_H_28_O_5_^37^Cl, 385.1590) (Supplementary Figures S13, S14).

**TABLE 1:** ^1^H (500 MHz) and ^13^C NMR (125 MHz) data for **1**.

	**1 (in DMSO-*d*_6_)**		**1 (in acetone-*d*_6_)**	
**Position**	**δ_C_, type**	**δ_H_, (*J* in Hz)**	**δ_C_, type**	**δ_H_, (*J* in Hz)**
1a	32.4, CH_2_	1.38-1.42, m	32.9, CH_2_	1.55-1.58, m
1b		1.29-1.36, m		1.48-1.54, m
2a	26.4, CH_2_	1.97, dt (13.5, 2.6)	26.9, CH_2_	2.08-2.17, m
2b		1.42-1.47, m		1.55-1.58, m
3	68.9, CH	3.76-3.80, m	70.2, CH	3.97-4.00, m
4	46.9, C		49.2, C	
5	47.6, CH	1.38-1.42, m	48.6, CH	1.41-1.47, m
6	22.2, CH_2_	1.53-1.66, m	22.8, CH_2_	1.72-1.78, m
7a	27.4, CH_2_	1.83-1.86, m	27.4, CH_2_	1.95-1.97, m
7b		0.90, dd (13.0, 4.6)		1.05-1.11, m
8	47.6, CH	1.29-1.36, m	48.3, CH	1.59-1.61, m
9	43.6, CH	1.42-1.47, m	44.4, CH	1.62-1.66, m
10	36.6, C		37.2, C	
11a	32.9, CH_2_	1.83-1.86, m	33.2, CH_2_	1.98-2.02, m
11b		1.05, dd (12.3, 2.6)		1.17-1.22, m
12	63.4, CH	4.94-4.97, m	64.6, CH	5.16, t (3.0)
13	147.2, C		147.2, C	
14	77.8, C		78.5, C	
15	141.0, CH	6.40, dd (17.2, 10.9)	141.5, CH	6.54, dd (17.2, 10.9)
16a	110.7, CH_2_	5.15, dd (17.2, 2.5)	110.5, CH_2_	5.25, dd (17.2, 2.3)
16b		4.90, dd (10.9, 2.5)		4.93, dd (10.9, 2.3)
17	112.8, CH	6.24, s	113.8, CH	6.36, s
18	24.3, CH_3_	1.10, s	24.3, CH_3_	1.25, s
19	178.8, C		178.8, C	
20	12.5, CH_3_	0.63, s	12.5, CH_3_	0.74, s
3-OH		4.45, d (4.4)		
12-OH		4.99, d (2.9)		
14-OH		4.69, s		
19-COOH		11.99 br s		

Crystal data for **1**: C_21_H_33_O_6_Cl, *M*r = 416.92, orthorhombic, *a* = 12.0987 (3) Å, *b* = 12.1802 (3) Å, *c* = 14.0007 (3) Å, α = 90.00°, β = 90.00°, γ = 90.00°, *V* = 2063.21 (8) Å^3^, space group *P*212121, *Z* = 4, *D*x = 1.342 mg/m^3^, μ (Cu Kα) = 1.934 mm^–1^, and *F* (000) = 896. Crystal dimensions: 0.24 mm × 0.22 mm × 0.20 mm. Independent reflections: 17532/3771 (*R*int = 0.0293). The final *R*1 value was 0.0286, *wR*2 = 0.0729 [*I* > 2σ(*I*)]. Flack parameter = 0.050(5) (Supplementary Data Sheet S2). Crystallographic data for **1** have been deposited in the Cambridge Crystallographic Data Center as supplementary publication number CCDC 1910127.

Harzianone E (**2**): Colorless oil; [α]${}_{\text{D}}^{27}$ + 21.5 (*c* 0.30, MeOH); UV (MeOH) λ_max_ (log ε) 206 (3.54) nm; ECD (3.16 μM, MeOH) λ_max_ (Δε) 254 (−3.90), 343 (+3.42) nm; IR (KBr) ν_max_ 3561, 2977, 1684, 1209, 1137, 842, 542 cm^–1^; ^1^H and ^13^C NMR data, see Table 2 and Supplementary Figures S15–S21; ESIMS *m/z* 339.3 [M + Na]^+^, 355.2 [M + K]^+^; HRESIMS *m/z* 339.1933 [M + Na]^+^ (calcd. for C_20_H_28_O_3_Na, 339.1931) (Supplementary Figures S22, S23).

**TABLE 2:** ^1^H (500 MHz) and ^13^C NMR (125 MHz) data for **2** and **3**.

	**2 (in CDCl_3_)**		**3 (in DMSO-*d*_6_)**	
**position**	**δ_C_, type**	**δ_H_, (*J* in Hz)**	**δ_C_, type**	**δ_H_, (*J* in Hz)**
1	46.4, C		15.1, CH_3_	0.88, d (6.7)
2	41.1, CH	1.94-1.98, m	43.7, CH	1.41–1.51, m
3	216.7, C		79.1, C	
4a	46.5, CH_2_	2.74, d (18.5)	40.5, CH_2_	1.41–1.51, m
4b		2.32-2.34, m		1.31–1.38, m
5a	46.2, CH	3.12, q (8.0)	23.9, CH_2_	1.57–1.67, m
5b				1.41–1.51, m
6	51.0, C		53.7, CH	1.57–1.67, m
7	29.8, CH_2_	1.27-1.32, m	72.9, C	
8a	24.2, CH_2_	2.26-2.28, m	41.6, CH_2_	1.21–1.30, m
8b		2.05-2.09, m		
9	153.0, C		18.5, CH_2_	1.21–1.30, m
10a	149.0, C		44.6, CH_2_	2.00–2.05, m
10b				1.21–1.30, m
11	199.3, C		68.8, C	
12a	59.1, CH_2_	2.58, d (17.0)	29.5, CH_3_	1.12, s
12b		2.48, d (17.0)		
13	39.4, C		26.3, CH_3_	1.08, s
14	53.0, CH	2.29, m	25.0, CH_3_	0.96, s
15a	29.7, CH_2_	2.09-2.13, m	29.2, CH_3_	1.04, s
15b		1.94-1.98, m		
16a	25.0, CH_3_	1.01, s		
17	22.2, CH_3_	1.13, s		
18	17.2, CH_3_	1.24, d (8.0)		
19	22.0, CH_3_	1.39, s		
20a	67.1, CH_2_	4.38, d (18.0)		
20b		4.25, dd (18.0, 6.6)		
3-OH				3.76, s
7-OH				3.74, s
11-OH				4.03, s
20-OH		4.53, s		

3,7,11-Trihydroxy-cycloneran (**3**): Colorless oil; [α]${}_{\text{D}}^{22}$ –29.9 (*c* 1.00, CHCl_3_); UV (MeOH) λ_max_ (log ε) 206 (4.05) nm; IR (KBr) ν_max_ 3673, 1684, 1540, 1457, 1382, 1209, 1139 cm^–1^; ^1^H and ^13^C NMR data, see Table 2 and Supplementary Figures S24–S30; ESIMS *m/z* 259.3 [M + H]^+^, 281.3 [M + Na]^+^; HRESIMS *m/z* 281.2095 [M + Na]^+^ (C_15_H_30_O_3_Na, calcd. for 281.2087) (Supplementary Figures S31, S32).

Crystal data for **12**: C_15_H_20_O_3_, *M*r = 248.31, orthorhombic, *a* = 8.3626 (3) Å, *b* = 10.8592 (4) Å, *c* = 14.9542 (5) Å, α = 90.00°, β = 90.00°, γ = 90.00°, *V* = 1358.01 (8) Å^3^, space group *P*212121, *Z* = 4, *D*x = 1.215 mg/m^3^, μ (Cu Kα) = 0.670 mm^–1^, and *F* (000) = 536. Crystal dimensions: 0.18 mm × 0.16 mm × 0.12 mm. Independent reflections: 19809/2479 (*R*int = 0.0285). The final *R*1 value was 0.0301, *wR*2 = 0.0833 [*I* > 2σ(*I*)]. Flack parameter = −0.05(5) (Supplementary Presentation S1). Crystallographic data for **12** have been deposited with the Cambridge Crystallographic Data Center as supplementary publication number CCDC 1910138.

### Antibacterial and Antifungal Activity Assay

The antibacterial and antifungal activity was evaluated by the conventional broth dilution assay (Appendino et al., 2008). Three pathogenic bacterial strains, *Staphylococcus aureus* (ATCC 27154), *Escherichia coli* (ATCC 25922) and *Pseudomonas aeruginosa* (ATCC 10145), six fouling bacterial strains *P. fulva* (ATCC 31418), *Aeromonas salmonicida* (ATCC 7965D), *Vibrio harveyi* (ATCC BAA-2752), *Photobacterium angustum* (ATCC 33975), *Enterobacter cloacae* (ATCC 39978) and *E. hormaechei* (ATCC 700323), and one pathogenic fungal strain *Candida albicans* (ATCC 76485) were used. Penicillin G was used as a positive control of pathogenic bacterial and fungal strains and Sea-Nine 211 was used as a positive control of fouling bacterial strains.

### DNA Topo I Inhibition Bioassay

The Topo I inhibitory activity was measured by assessing the relaxation of supercoiled pBR322 plasmid DNA (Bogurcu et al., 2011). Camptothecin (CPT) was used as a positive control. The gel was stained with Gelred and visualized under UV illumination and then photographed with a Gel imaging system.

### AChE Inhibition Bioassay

The AChE inhibition activity was measured based on the modified Ellman’s method (Ellman et al., 1961). Huperzine A and galanthamine were used as positive drugs. The inhibition rates of AChE were calculated using Origin 8.0 software.

## Results

### Chemical Identification of the Isolated Compounds

**FIGURE 1:**
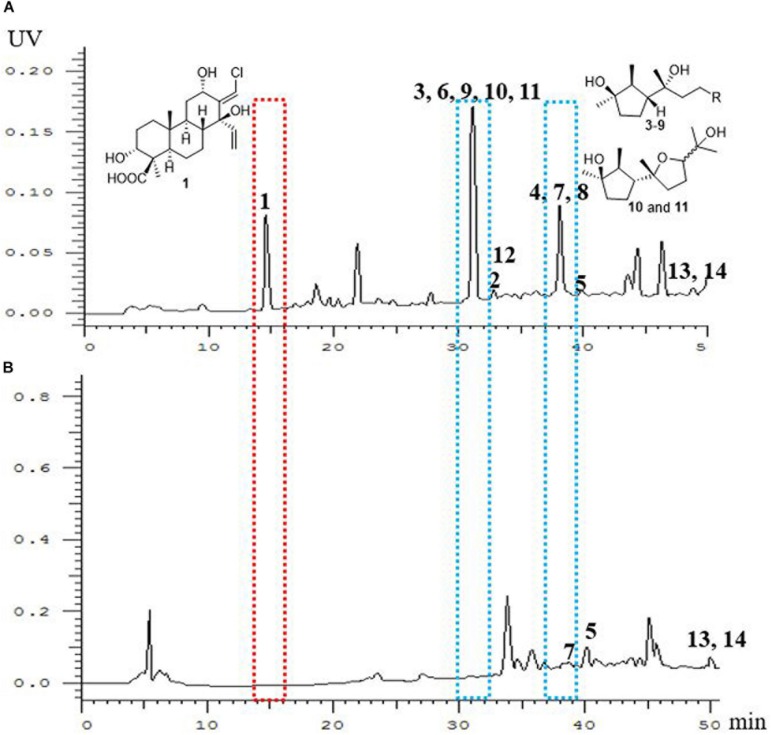
HPLC profiles of EtOAc extracts of *T. harzianum* (XS-20090075) cultivated in rice medium. **(A)** treated with 10 μM sodium butyrate; **(B)** the control; HPLC chromatograms: C_18_ column using a gradient of 5–100% MeOH in H_2_O. The peak in the red dotted box means new obtained compound cleistanthane diterpenoid. The peaks in the blue dotted boxes means main metabolic products.

**FIGURE 2:**
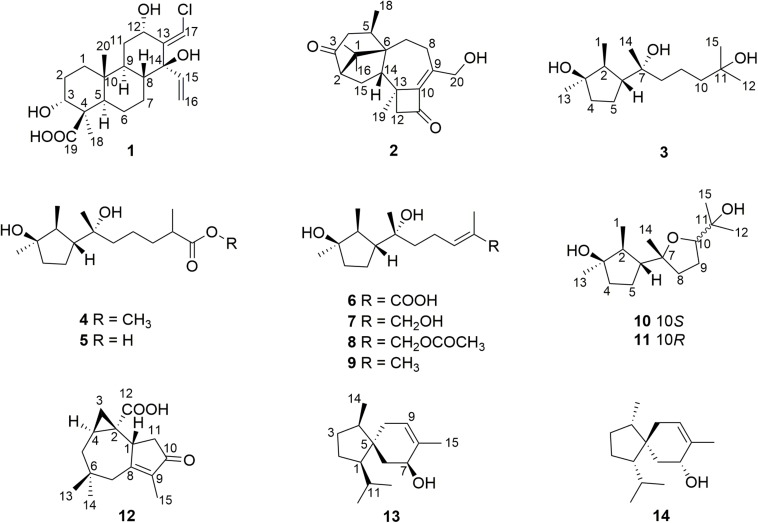
Chemical structures of **1**–**14**.

The chemical epigenetic modification was conducted on the fungus *T. harzianum* (XS-20090075) by adding HDAC and DNA methytransferase (DNMT) inhibitors with different concentrations (Supplementary Figure S1). The results showed that a HDAC inhibitor, sodium butyrate, induced significant changes of the fungal metabolic profile. Compared to the rice culture control at the same culture condition, the HPLC finger-print of the EtOAc extract of the culture with sodium butyrate (10 μM) in rice medium showed new peaks at about 15, 31, and 38 min (Figure 1A), and the main peaks of the harziane diterpenoids at 33–42 min (Figure 1B) disappeared. Chemical investigation of the EtOAc extract led to the isolation of three new terpenoids, including one novel chlorinated cleistanthane diterpenoid, harzianolic acid A (**1**), one harziane diterpenoid, harzianone E (**2**), and one cyclonerane sesquiterpenoid, 3,7,11-trihydroxy-cycloneran (**3**), together with 11 known sesquiterpenoids, including eight cyclonerane sesquiterpenoids, methyl 3,7-dihydroxy-15-cycloneranate (**4**) (Song et al., 2018), catenioblin C (**5**) (Wu et al., 2012), ascotrichic acid (**6**) (Xie et al., 2013), cyclonerotriol (**7**) (Kasitu et al., 1992), (10*E*)-12-acetoxy-10-cycloneren-3,7-diol (**8**) (Fang et al., 2018), cyclonerodiol (**9**) (Nozoe et al., 1970), cyclonerodiol oxide (**10**) (Fujita et al., 1984) and epicyclonerodiol oxide (**11**) (Fujita et al., 1984), one african sesquiterpenoid, ophioceric acid (**12**) (Reátegui et al., 2005), and two acorane-type sesqiuterpenoids, *ent*-trichoacorenol (**13**) (Brock and Dickschat, 2011) and trichoacorenol (**14**) (Huang et al., 1995) (Figure 2). These results revealed that the original main products harziane diterpenoids were replaced by cyclonerane sesquiterpenoids. More importantly, newly produced metabolites cleistanthane diterpenoids and african sesquiterpenoids were uncovered from *T. harzianum* (XS-20090075) by epigenetic modifying treatments.

**FIGURE 3:**
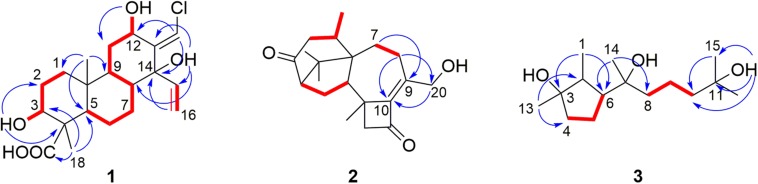
Key HMBC (blue arrows) and COSY (red bold lines) correlations of **1**–**3**.

Harzianolic acid (**1**) was obtained as yellow, needle crystals. The molecular formula C_20_H_29_O_5_Cl of **1** was determined by HRESIMS spectrum, indicating six degrees of unsaturation. The IR spectrum showed hydroxyl (3445 cm^–1^) and carboxyl (2921, 1700 cm^–1^) characterized absorption bands. The ^1^H NMR, ^13^C NMR (Table 1) and HSQC spectra of **1** exhibited two methyl groups, six methylene groups including one olefinic-methylene, seven methines including two olefinic-methines and two oxy-methines, and five non-protonated carbons including one carboxyl group and one olefinic carbon. These NMR signals combined with its degrees of unsaturation indicated that **1** should be a tricyclic diterpenoid belonging to the family of cleistanthane-type diterpenoid. The spectroscopic feature of **1** was similar to those of zythiostromic acid B which was isolated from the fungus *Zythiostroma* sp. derived from aspen *Populus tremuloides* Michx (Ayer and Khan, 1996). However, the hydroxyl substituent at C-5 in zythiostromic acid B was replaced by a hydrogen atom in **1**, indicating by the methine signal of H-5 in ^1^H NMR and the upfield of C-5 in ^13^C NMR. This was also confirmed by the COSY cross peak of H-5/H-6 and HMBC correlations from H-18 and H-20 to C-5 (Figure 3). The COSY correlation of H-12/12-OH, HMBC correlation from 12-OH to C-11 and the downfield shift of C-12 in ^13^C NMR indicated a hydroxyl group locating at C-12. The cross peaks from 14-OH to C-8, C-13, C-14, C-15 in HMBC, as well as the downfield shift of C-14 in ^13^C NMR suggested a hydroxyl group anchoring at C-14. The ESIMS of **1** exhibited chlorine isotope peak ratio of 3:1 revealed that a chlorine substitute should occur in **1**. The methylene at C-17 in zythiostromic acid B was replaced by the methine in **1**, indicating the chlorine was located at C-17, which was confirmed by the downfield shifts of C-17 in ^13^C NMR and H-17 in ^1^H NMR.

**FIGURE 4:**
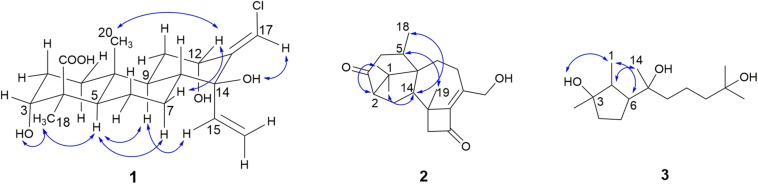
Key NOESY correlations of **1**–**3**.

The relative configuration of **1** was determined by NOESY data. The NOESY spectrum of **1** measured in DMSO-*d*_6_ was not enough to indicate its relative configuration (Supplementary Figure S8). So it was measured again in acetone-*d*_6_ (Supplementary Figure S12). The NOESY correlations of H-5/H-7b, H-5/H-9, and H-5/H-18, and the correlations of H-9/H-15 and H-18/3-OH indicated that H-5, H-7b, H-9, H-15, H-18, and 3-OH were in the same face (Figure 4). The correlations of H-12/H-7a and H-12/H-20 suggested H-7a, H-12, and H-20 were in another face (Figure 4). The NOESY correlation between H-17 and 14-OH indicated the *E* configuration of the double bond at C-13 and C-17 (Figure 4).

**FIGURE 5:**
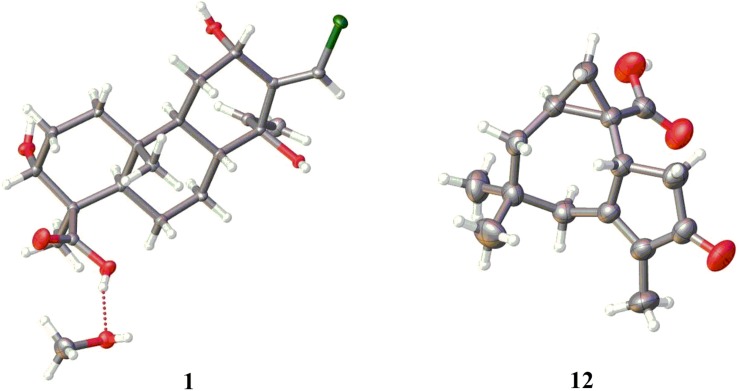
Single crystal X-ray structures of **1** and **12**.

The absolute configuration of **1** was first attempted to solve by the modified Mosher’s method. Unfortunately, this method was unsuccessful due to the limited quantity of **1** and the multiple hydroxyls in **1** resulting in the complex products. Fortunately, the single crystals of **1** suitable for X-ray diffraction analysis was obtained by slowly crystallization from the mixture solvent of CH_3_OH/CH_2_Cl_2_/H_2_O (20:20:1). The stereochemistry of **1** was undisputed confirmed to be 3*R*,4*R*,5*R*,8*R*,9*S*,10*R*,12*S*,14*S*,13*E* by Cu Kα X-ray diffraction with a Flack’s parameter of 0.050(5) (Figure 5).

Harzianone E (**2**) was obtained as a colorless oil. The molecular formula of C_20_H_28_O_3_ was determined by HRESIMS indicating seven degrees of unsaturation. The IR absorption bands at 3561 and 1685 cm^–1^ indicated the presence of hydroxyl and carbonyl groups. The analyzing of ^1^H NMR and ^13^C NMR spectra of **2** (Table 2) suggested that **2** belonged to the family of harziane diterpenoid and was very similar to harziandione which was first isolated from the biological control agent *T. harzianum* Rifai (Ghisalberti et al., 1992). The only difference between **2** and harziandione was the oxy-methylene (δ_C_ 67.1, δ_H_ 4.38) at C-20 in **2** instead of the methyl (δ_C_ 22.5, δ_H_ 2.12) at C-20 in harziandione, revealing a hydroxyl substituent at C-20. This was confirmed by the correlations from H-8b to C-20, and from H-20 to C-10 in HMBC of **2** (Figure 3). The relative configuration of **2** was determined by NOESY data. The NOESY correlations of H-16/H-14, H-17/H-2, and H-18/H-14 indicated that H-2, H-14, H-16, H-17, and H-18 were in the same face (Figure 4). The NOESY correlation between H-5 and H-19 suggested that H-5 and H-19 were located on another face of the molecule (Figure 4). Thus, the relative configuration of **2** was determined as 2*S*^∗^,5*R*^∗^,6*R*^∗^,13*S*^∗^,14*S*^∗^.

**FIGURE 6:**
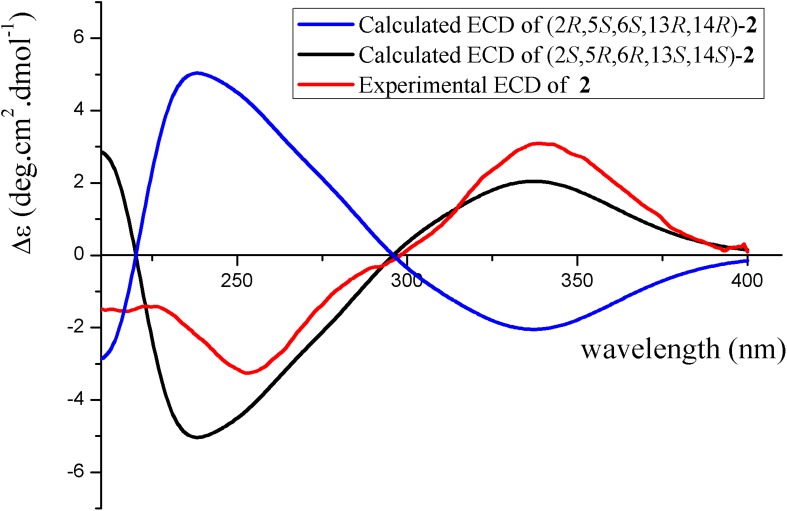
Experimental and calculated ECD spectra of **2**.

The absolute configuration of **2** was determined by theoretical calculated electronic circular dichroism (ECD) and optical rotation (OR). The conformations of (2*S*,5*R*,6*R*,13*S*,14*S*)-**2** and (2*R*,5*S*,6*S*,13*R*,14*R*)-**2** were searched through the MMFF94S method. The results both exhibited 5 lowest energy conformers with relative energies from 0 to 10 kcal/mol. The set of gas-phase B3LYP/6-31G(d) level was used for the first optimization carried out by Gaussian 09 package, both resulting in 2 conformers whose relative energies were within 4.6 kcal/mol. The conformers were re-optimized using the set of gas-phase B3LYP/6-311+G(d) and then were calculated ECD and OR at the set of gas-phase B3LYP/6-311++g(2d,p). Boltzmann statistics were used to simulated ECD curves with a standard deviation of 0.16 eV. The experimental ECD spectrum of **2** showed first negative (254 nm) and second positive (343 nm) Cotton effects, matching well with the theoretical ECD spectrum for (2*S*,5*R*,6*R*,13*S*,14*S*)-**2** between 200 and 400 nm (Figure 6), which determined the absolute configuration of **2**. The experimental OR ([α]${}_{\text{D}}^{27}$ + 21.5 (*c* 0.30, MeOH)) was comparative to the calculated OR of (2*S*,5*R*,6*R*,13*S*,14*S*)-**2** (+ 46.1) confirmed the absolute configuration of **2**.

3,7,11-Trihydroxy-cycloneran (**3**) was also isolated as a colorless oil. Its molecular formula was determined as C_15_H_30_O_3_ based on HRESIMS, requiring one degree of unsaturation. The ^1^H NMR, ^13^C NMR (Table 2) and HSQC analysis, combining with its degree of unsaturation, suggested that **3** belonged to the family of monocycle sesquiterpenoid and was very similar to cyclonerodiol (**9**) which was first isolated from a *Trichothesium* sp. Fungus (Fang et al., 2018). The double bond signals at C-10 (δ_C_ 124.6, δ_H_ 5.12) and C-11 (δ_C_ 132.0) in **9** were absent in **3** and were replaced by a methylene (δ_C_ 44.6, δ_H_ 2.00–2.05, 1.21–1.31) and an oxygen-bearing non-protonated carbon (δ_C_ 68.8). The COSY cross peak of H-9/H-10, and HMBC correlations from 11-OH to C-10, C-11, and C-15 (Figure 3) confirmed the above deduction. The relative configuration of **3** was determined by NOESY data. The *syn* relationship of H-1, H-6, and 3-OH was deduced by the NOESY cross peak of H-1/H-6 and H-1/3-OH (Figure 4). The correlation between H-2 and H-14 in NOESY indicated that H-2 and H-14 lied on the same side (Figure 4). It is difficult to determine the absolute configuration of C-7 in **3** due to the rotated single bond between C-6 and C-7. Mosher’s method (Cao et al., 2014) was tried to determine the absolute configuration of C-7 but failed. Fortunately, a literature survey revealed that the co-isolated cyclonerane-type compounds (**4**–**9**) and many other similar natural compounds have been reported the same absolute configurations as 2*S*,3*R*,6*R*,7*R* (Hanson et al., 1975; Kasitu et al., 1992; Li et al., 2007; Wu et al., 2012; Xie et al., 2013; Zhang et al., 2017; Fang et al., 2018; Song et al., 2018). Therefore, based on the biogenetic considerations, compound **3** was proposed to have the same absolute configuration (2*S*,3*R*,6*R*,7*R*) as the co-isolated compound **9**, which was also supported by their similar OR between **3** ([α]${}_{\text{D}}^{22}$ –29.9 (*c* 1.00, CHCl_3_)) and **9** ([α]${}_{\text{D}}^{22}$ –26.6 (*c* 1.00, CHCl_3_); lit. [α]${}_{\text{D}}^{\text{rrt}}$ –20.0 (*c* 0.76, CHCl_3_) (Fang et al., 2018)).

Compounds **4**–**14** were identified to be methyl 3,7-dihydroxy-15-cycloneranate (Song et al., 2018), catenioblin C (Wu et al., 2012), ascotrichic acid (Xie et al., 2013), cyclonerotriol (Kasitu et al., 1992), (10*E*)-12-acetoxy-10-cycloneren-3,7-diol (Fang et al., 2018), cyclonerodiol (Nozoe et al., 1970), cyclonerodiol oxide (Fujita et al., 1984), epicyclonerodiol oxide (Fujita et al., 1984), ophioceric acid (Reátegui et al., 2005), *ent*-trichoacorenol (Brock and Dickschat, 2011), and trichoacorenol (Huang et al., 1995), respectively, by comparing their NMR data with those in the literature. The absolute configuration of **12** (1*R*,2*S*,4*S*) was confirmed for the first time using the method of single X-ray diffraction with the Flack’s parameter of −0.05(5) (Figure 5).

### Plausible Biogenetic Pathways Proposed for 1–14

**FIGURE 7:**
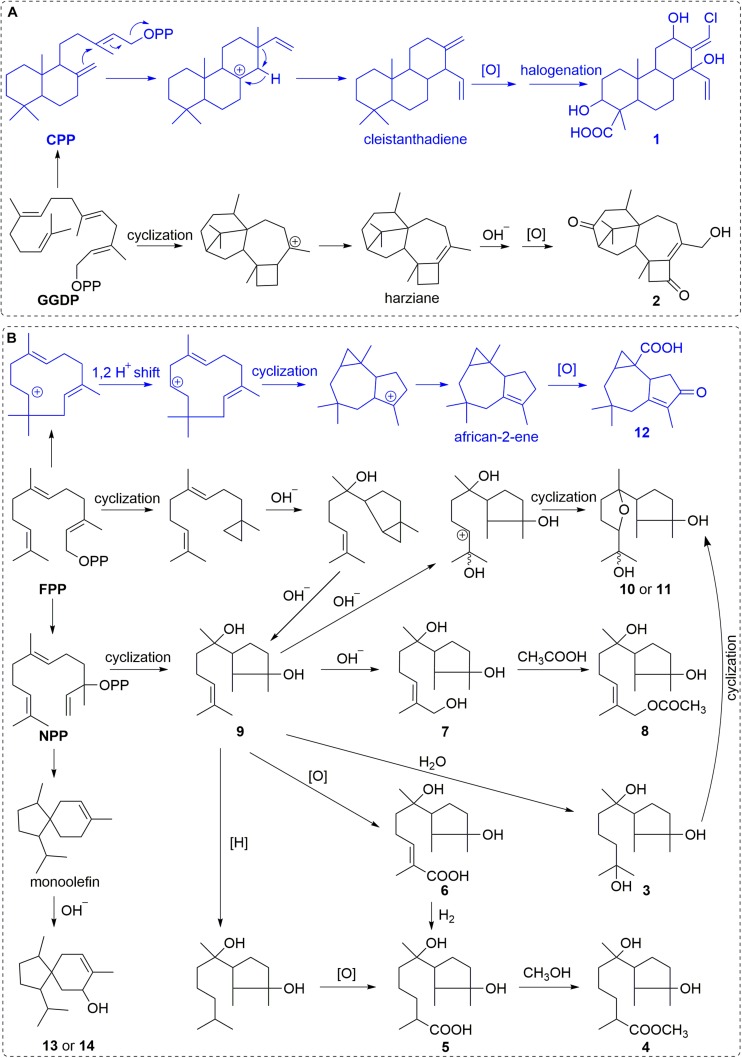
The plausible biogenetic pathways proposed for **1–2 (A)** and **3–14 (B)**.

The emerged main metabolic pathway of *T. harzianum* (XS20090075) was the biosynthesis of harziane diterpens at the traditional experimental condition in rice culture (Zhao et al., 2019). By chemical epigenetic manipulation, the biosynthesis of harziane diterpens was depressed. Only one harziane diterpen, harzianone E (**2**), was obtained in this study, which might be the hydroxylation and oxidation derivative of harziane (Figure 7A) (Adelin et al., 2014). Absorbingly, the new produced cleistanthane diterpenoid, harzianolic acid A (**1**), might reveal the activation of cleistanthane diterpenoid biosynthesis pathway. Compound **1** may be generated from CPP to cleistanthadiene by cyclization and rearrangement (Bai et al., 2018), followed by the oxidation and halogenation of cleistanthadiene (Figure 7A). In contrast, the biosynthetic pathway of cyclonerane sesquiterpenoids was activated, resulting in the discovery of a series of cyclonerane sesquiterpenoids, 3,7,11-trihydroxy-cycloneran (**3**) and **4**–**11**. The biosynthesis pathway of these sesquiterpenoids may start from FPP or NPP to **9** through cyclization (Evans et al., 1976), followed by oxidation, hydroxylation, hydration or reduction procedures (Figure 7B). Interestingly, the new produced ophioceric acid (**12**) might reveal the activation of the biosynthesis of african sesquiterpenoids due to epigenetic manipulation. Compound **12** may be produced from FPP to african-2-ene through cyclization (Wawrzyn et al., 2012), followed by oxidation reaction (Figure 7B).

### Bioactivities of Compounds 1–14

All of the isolated compounds (**1**–**14**) were evaluated for their antibacterial, antifungal, DNA topoisomerase I (Topo I) inhibitory and acetylcholinesterase (AChE) inhibitory activities. The results indicated that only **2** and **4** exhibited weak antibacterial activity against *P. angustum*.

## Discussion

Our continuing research on the metabolic products from the fungal strain *T. harzianum* (XS-20090075) revealed the presence of the main products, harziane diterpenoids, in rice culture (Zhao et al., 2019). Attractively, the metabolic profiles of the fungal strain have been changed significantly after chemical epigenetic manipulation, resulting in the emergence of cyclonerane sesquiterpenoids as main metabolic products and the restraining of the production of harziane diterpenoids. Interestingly, a new type of diterpenoid, cleistanthane diterpenoid, arisen owing to epigenetic modification. It should be noted that the cleistanthane diterpenoid and african sesquiterpenoid were the first time to be discovered from *Trichoderma* species and this was the first time to discover cleistanthane diterpenoid containing chlorine.

This study was the first time to regulate the secondary metabolites of fungus *T. harzianum* applying chemical epigenetic manipulation, leading to the significant changes of its metabolic profiles. Evidently, this attempt will offer a new approach to mine the secondary metabolites from *T. harzianum*. Furthermore, this research was also a powerful evidence to prove that chemical epigenetic manipulation could be an efficient way to wake the silence genes of marine-derived fungi to discover new natural compounds, and even to promote the research and development of marine natural products.

## Conclusion

In summary, the chemical epigenetic manipulation of the soft coral-derived fungus *T. harzianum* (XS-20090075) led to the significant changes of its metabolic profiles. The epigenetic modification suppressed the biosynthesis pathway of harziane diterpenoids while excited the pathway of cyclonerane sesquiterpenoids, resulting in the alteration of main metabolic products from harziane diterpenoids to cyclonerane sesquiterpenoids (**3**–**11**). Intriguingly, two new biosynthesis pathway of cleistanthane diterpenoid and african sesquiterpenoid were found to be activated by epigenetic modification, illustrated by the appearance of cleistanthane diterpenoid harzianolic acid A (**1**) and african sesquiterpenoid ophioceric acid (**12**). Noticeably, the cleistanthane diterpenoids and african sesquiterpenoids were the first time to be discovered from *Trichoderma* and this was the first time to discover cleistanthane diterpenoid containing chlorine. These results demonstrated that the chemical epigenetic manipulation should be an efficient technique for the discovery of new secondary metabolites from marine-derived fungi.

## Data Availability Statement

The datasets generated for this study can be found in the GenBank(NCBI) accession number KU866299; The CCDC number of crystals of **1** was 1910127, **12** was 1910138.

## Author Contributions

TS contributed to the fermentation, extraction, isolation, and manuscript preparation. C-LS, YL, and D-LZ reviewed and amended the manuscript. FC contributed to the quantum chemistry calculation. X-MF, J-YY, J-SW, and Z-KZ contributed to the bioactivities test. C-YW was the project leader, organized and guided the experiments and manuscript writing.

## Conflict of Interest

The authors declare that the research was conducted in the absence of any commercial or financial relationships that could be construed as a potential conflict of interest.

## Supplementary Material

DATA SHEET S2 Crystal X-ray data of **1** and **12**.
