# Supplementary material for: Terpenoids From the Coral-Derived Fungus Trichoderma harzianum (XS-20090075) Induced by Chemical Epigenetic Manipulation
Source: Front Microbiol. 2020 Apr 3;11:572. doi: 10.3389/fmicb.2020.00572 (PMC7147461; doi:10.3389/fmicb.2020.00572)
Supplement: Supplementary file 1 [file Data_Sheet_1.docx]

Supplementary Material

**Figure S1.** HPLC profiles of EtOAc extracts of *T. harzianum* (XS-20090075) cultivated in rice medium. HPLC chromatograms: C_18_ column using a gradient of 5–100% MeOH in H_2_O.

**Figure S2.** ^1^H NMR (500 MHz, DMSO-*d*_6_) spectrum of compound **1**

**Figure S3.** Partial ^1^H NMR (500M Hz, DMSO-*d*_6_) spectrum of compound **1**

**Figure S4.** ^13^C NMR (500 MHz, DMSO-*d*_6_) spectrum of compound **1**

**Figure S5.** HSQC (DMSO-*d*_6_) spectrum of compound **1**

**Figure S6.** COSY (DMSO-*d*_6_) spectrum of compound **1**

**Figure S7.** HMBC (DMSO-*d*_6_) spectrum of compound **1**

**Figure S8.** NOESY (DMSO-*d*_6_) spectrum of compound **1**

**Figure S9.** ^1^H NMR (500 MHz, acetone-*d*_6_) spectrum of compound **1**

**Figure S10.** ^13^C NMR (500 MHz, acetone-*d*_6_) spectrum of compound **1**

**Figure S11.** HSQC (acetone-*d*_6_) spectrum of compound **1**

**Figure S12.** NOESY (acetone-*d*_6_) spectrum of compound **1**

**Figure S13.** ESIMS spectrum of compound **1**

**Figure S14.** HRESIMS spectrum of compound **1**

**Figure S15.** ^1^H NMR (500 MHz, CDCl_3_) spectrum of compound **2**

**Figure S16.** Partial ^1^H NMR (500 MHz, CDCl_3_) spectrum of compound **2**

**Figure S17.** ^13^C NMR (500 MHz, CDCl_3_) spectrum of compound **2**

**Figure S18.** HSQC (CDCl_3_) spectrum of compound **2**

**Figure S19.** COSY (CDCl_3_) spectrum of compound **2**

**Figure S20.** HMBC (CDCl_3_) spectrum of compound **2**

**Figure S21.** NOESY (CDCl_3_) spectrum of compound **2**

**Figure S22.** ESIMS spectrum of compound **2**

**Figure S23.** HRESIMS spectrum of compound **2**

**Figure S24.** ^1^H NMR (500 MHz, DMSO-*d*_6_) spectrum of compound **3**

**Figure S25.** Partial ^1^H NMR (500 MHz, DMSO-*d*_6_) spectrum of compound **3**

**Figure S26.** ^13^C NMR (500 MHz, DMSO-*d*_6_) spectrum of compound **3**

**Figure S27.** HSQC (DMSO-*d*_6_) spectrum of compound **3**

**Figure S28.** COSY (DMSO-*d*_6_) spectrum of compound **3**

**Figure S29.** HMBC (DMSO-*d*_6_) spectrum of compound **3**

**Figure S30.** NOESY (DMSO-*d*_6_) spectrum of compound **3**

**Figure S31.** ESIMS spectrum of compound **3**

**Figure S32.** HRESIMS spectrum of compound **3**


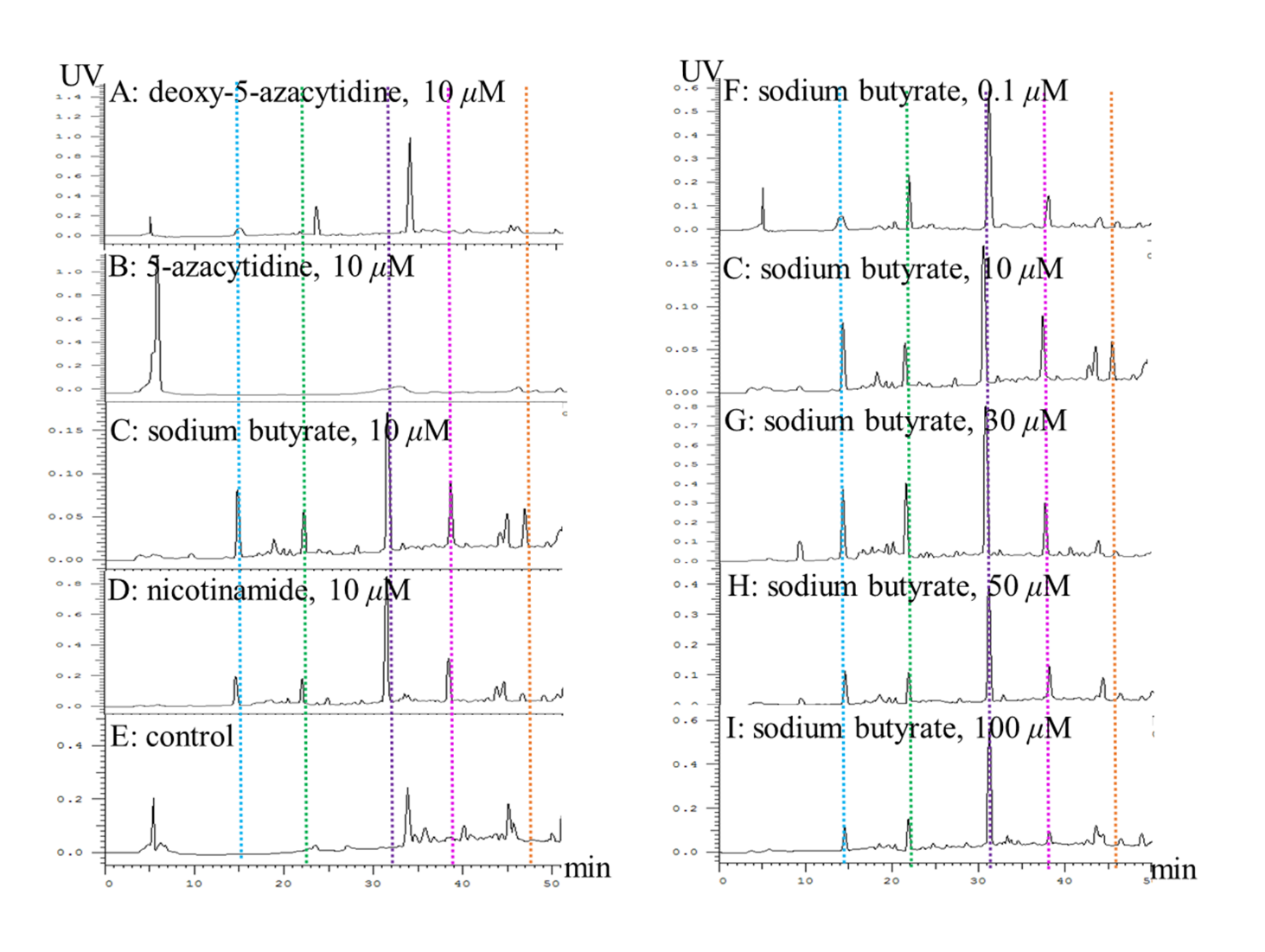


**Figure S1.** HPLC profiles of EtOAc extracts of *T. harzianum* (XS-20090075) cultivated in rice medium. HPLC chromatograms: C_18_ column using a gradient of 5–100% MeOH in H_2_O.


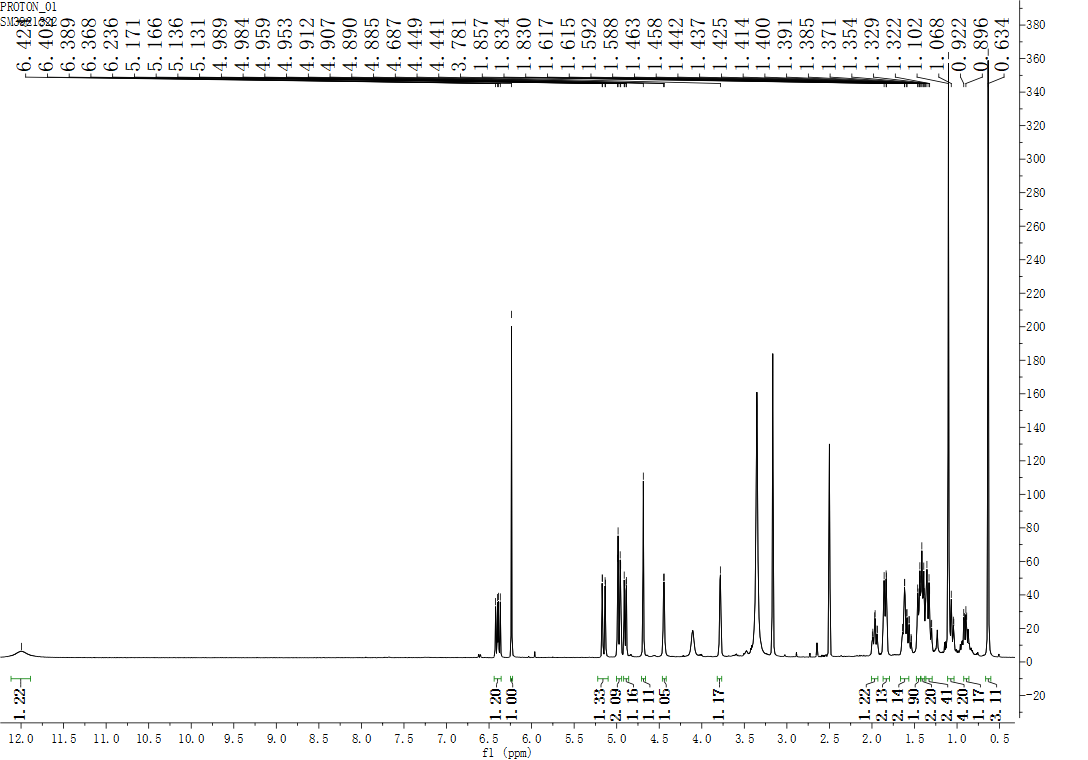


**Figure S2.** ^1^H NMR (500M Hz, DMSO-*d*_6_) spectrum of compound **1**

**
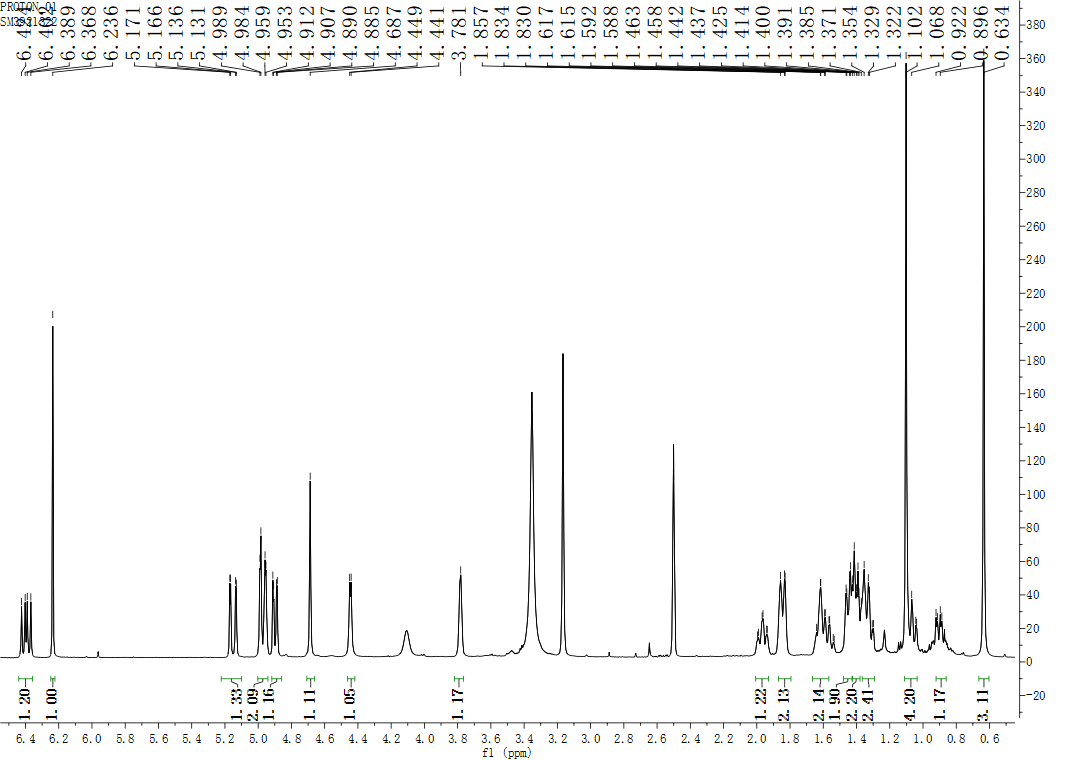
**

**Figure S3.** Partial ^1^H NMR (500M Hz, DMSO-*d*_6_) spectrum of compound **1**


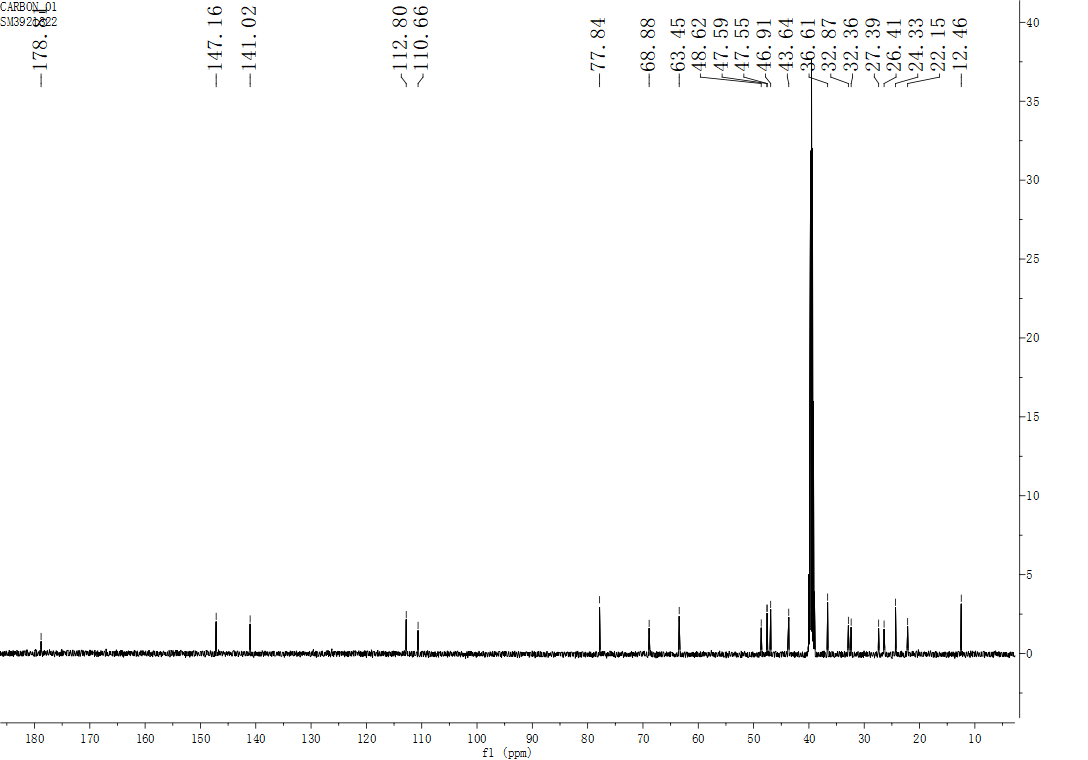


**Figure S4.** ^13^C NMR (125M Hz, DMSO-*d*_6_) spectrum of compound **1**


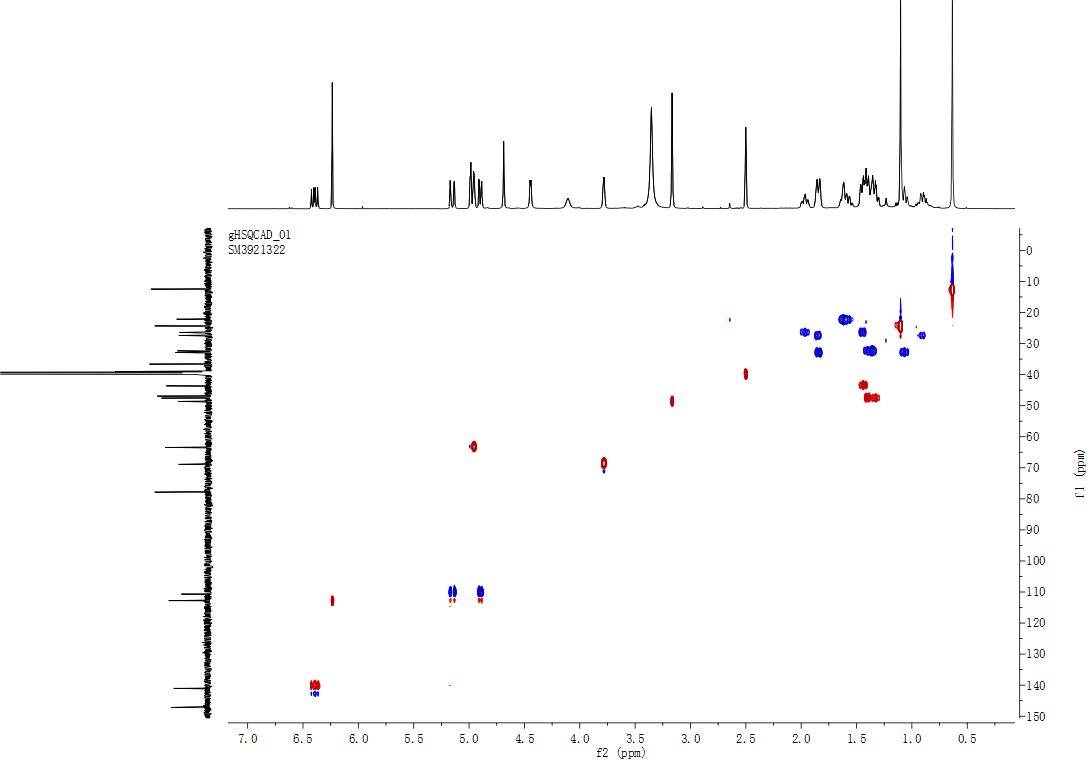


**Figure S5.** HSQC (DMSO-*d*_6_) spectrum of compound **1**


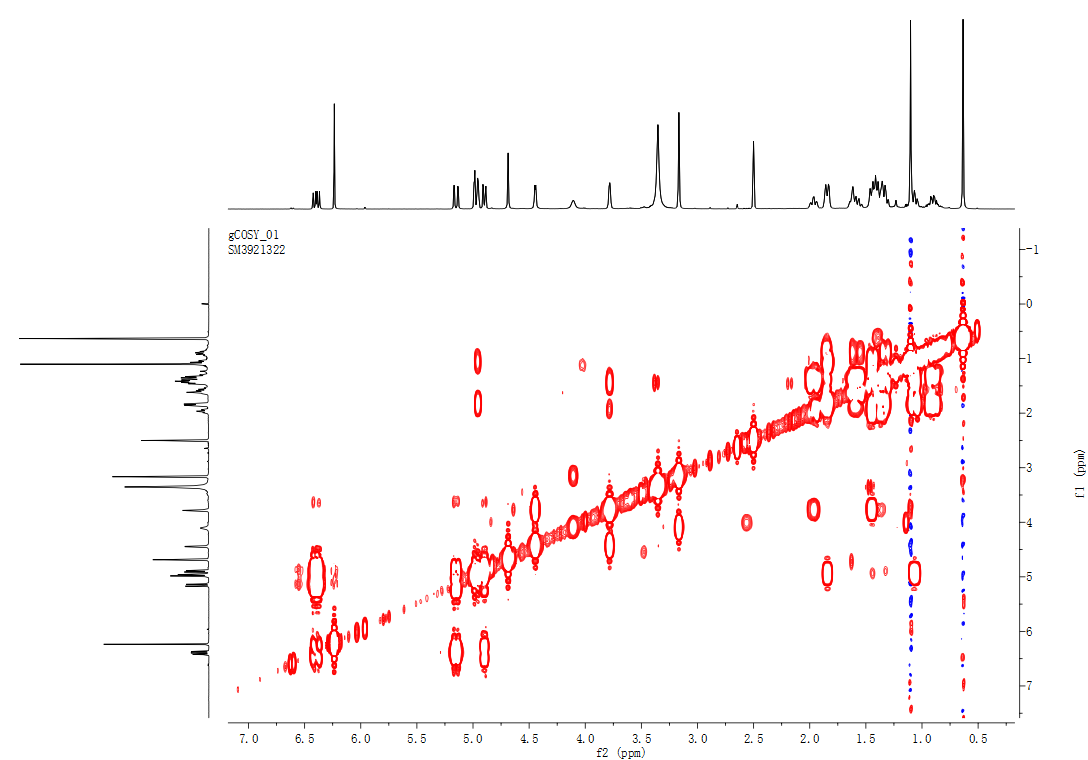


**Figure S6.** COSY (DMSO-*d*_6_) spectrum of compound **1**


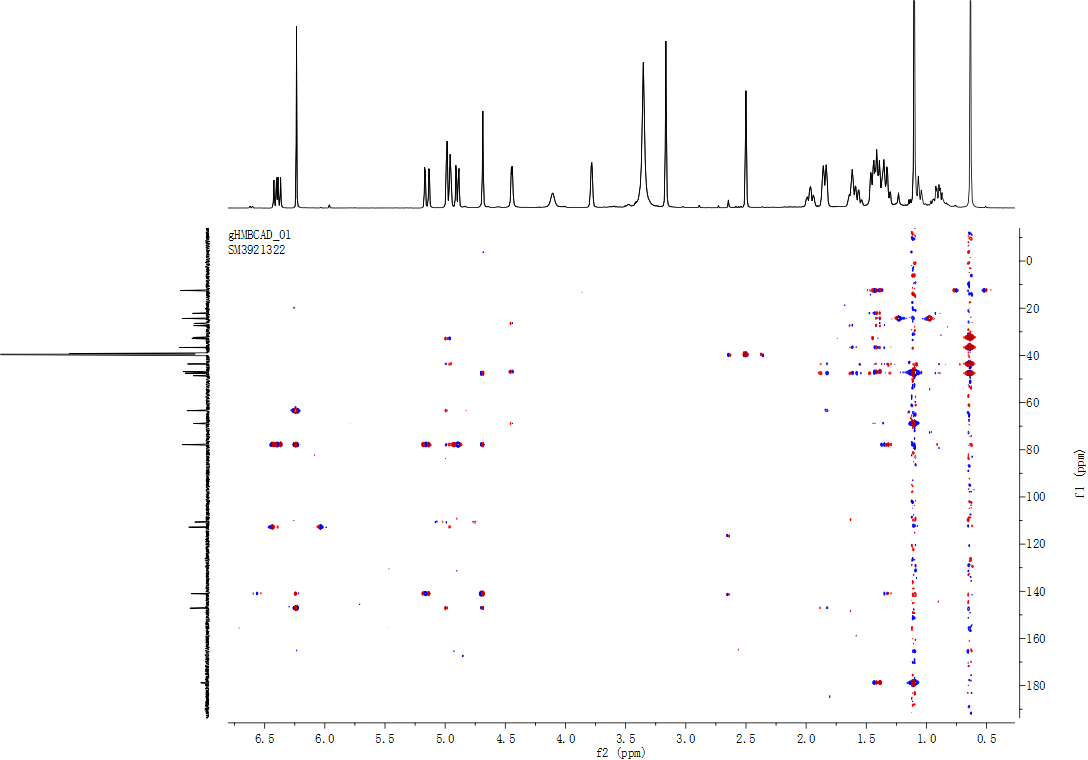


**Figure S7.** HMBC (DMSO-*d*_6_) spectrum of compound **1**


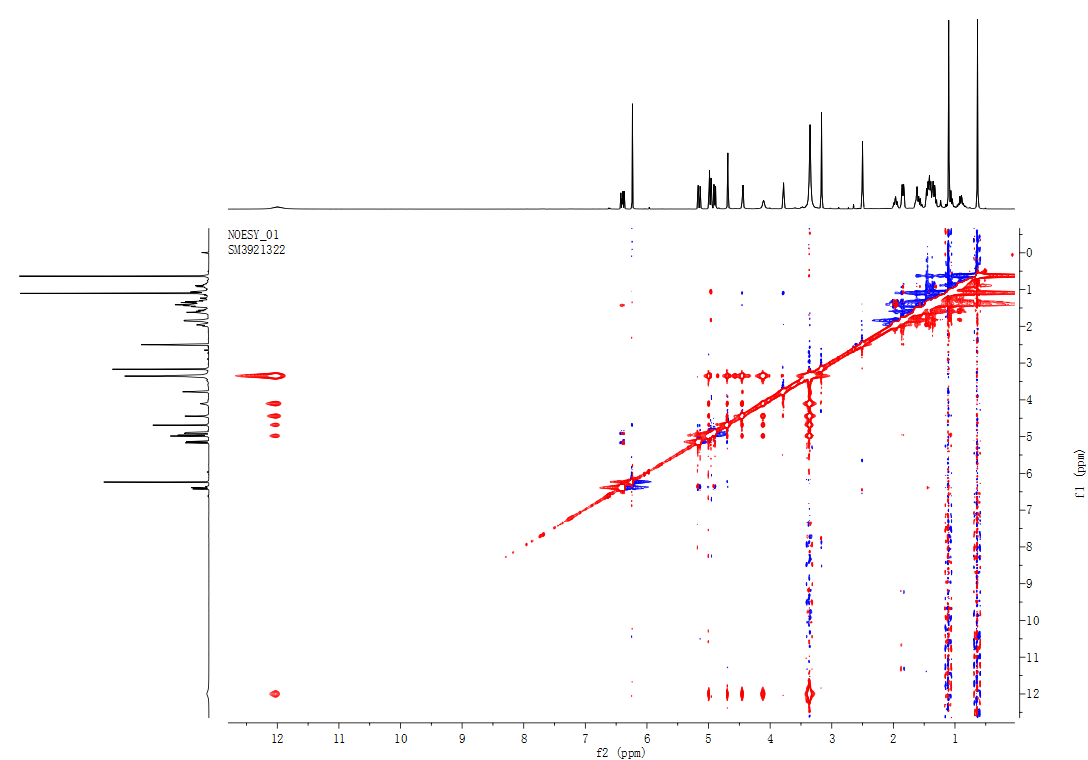


**Figure S8.** NOESY (DMSO-*d*_6_) spectrum of compound **1**


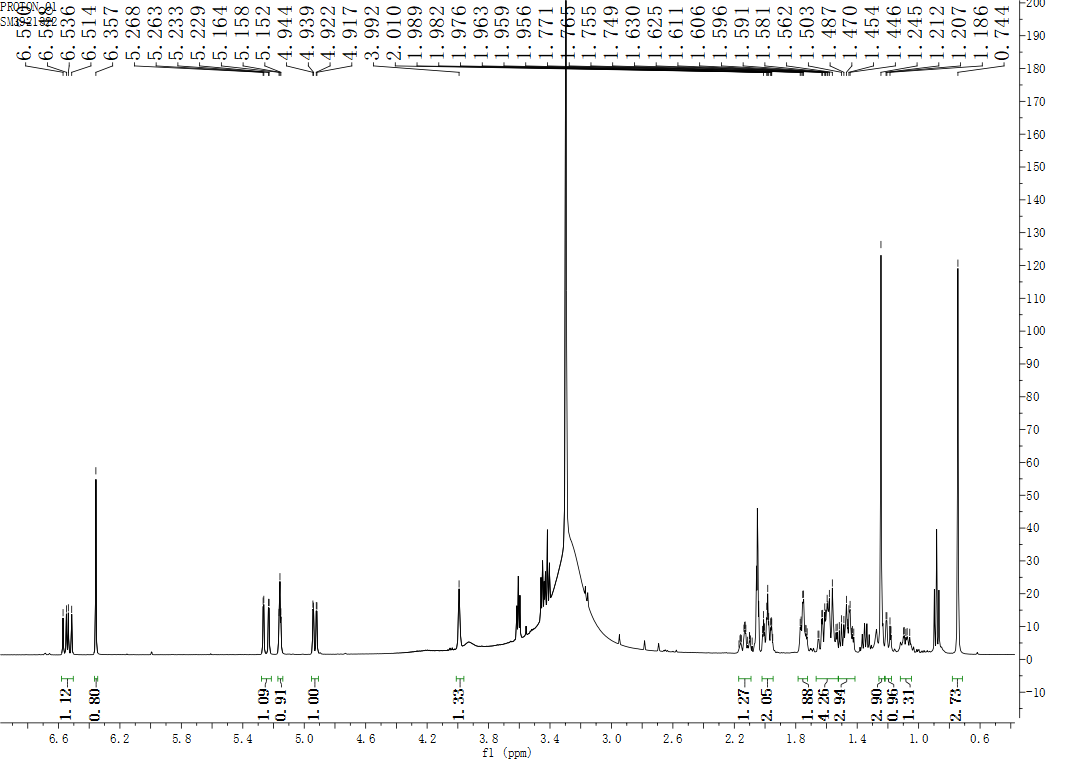


**Figure S9.** ^1^H NMR (500M Hz, acetone-*d*_6_) spectrum of compound **1**


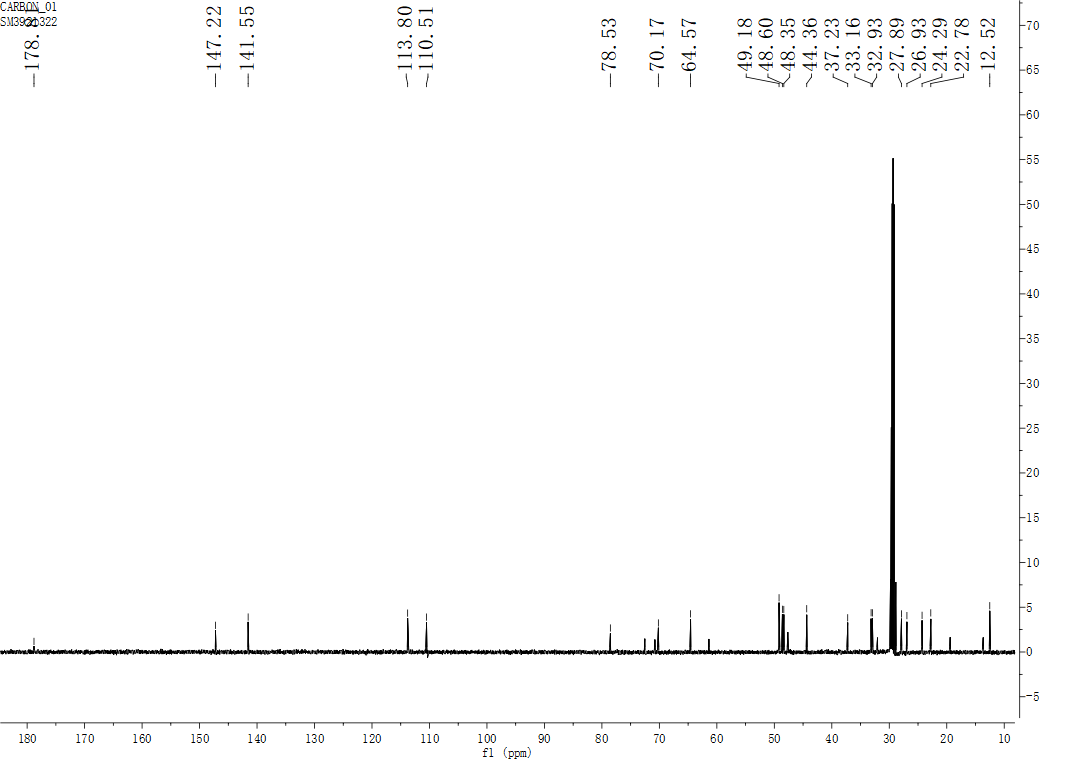


**Figure S10.** ^13^C NMR (500M Hz, acetone-*d*_6_) spectrum of compound **1**


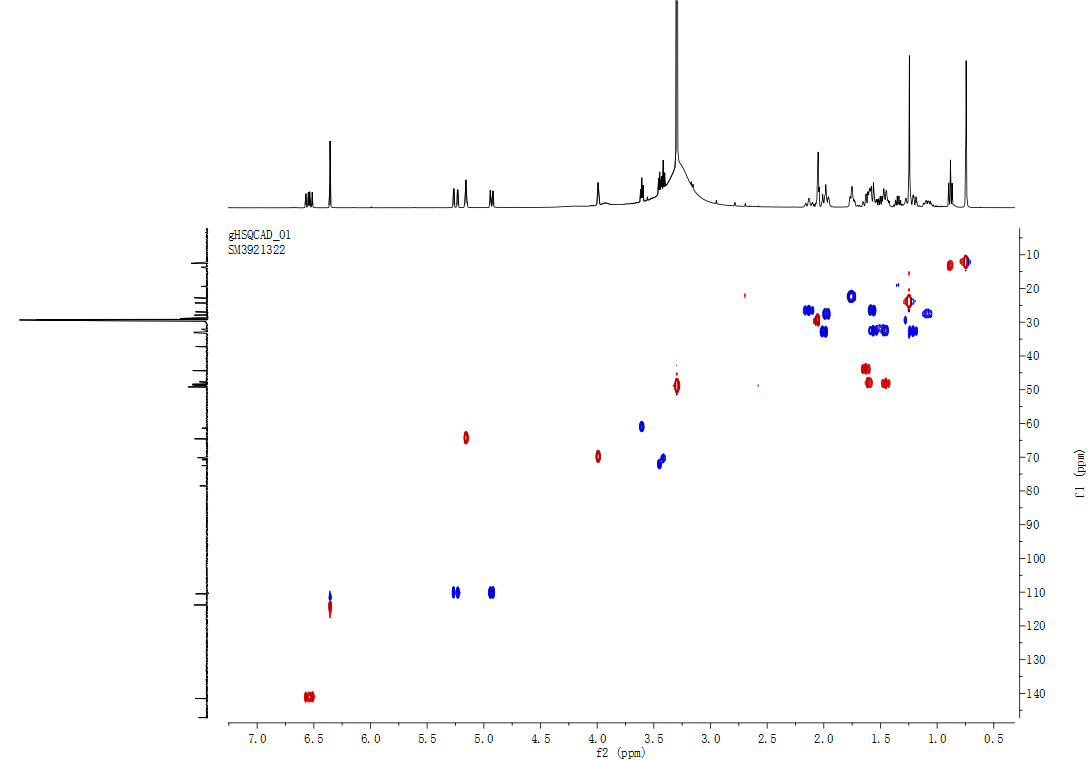


**Figure S11.** HSQC (acetone-*d*_6_) spectrum of compound **1**


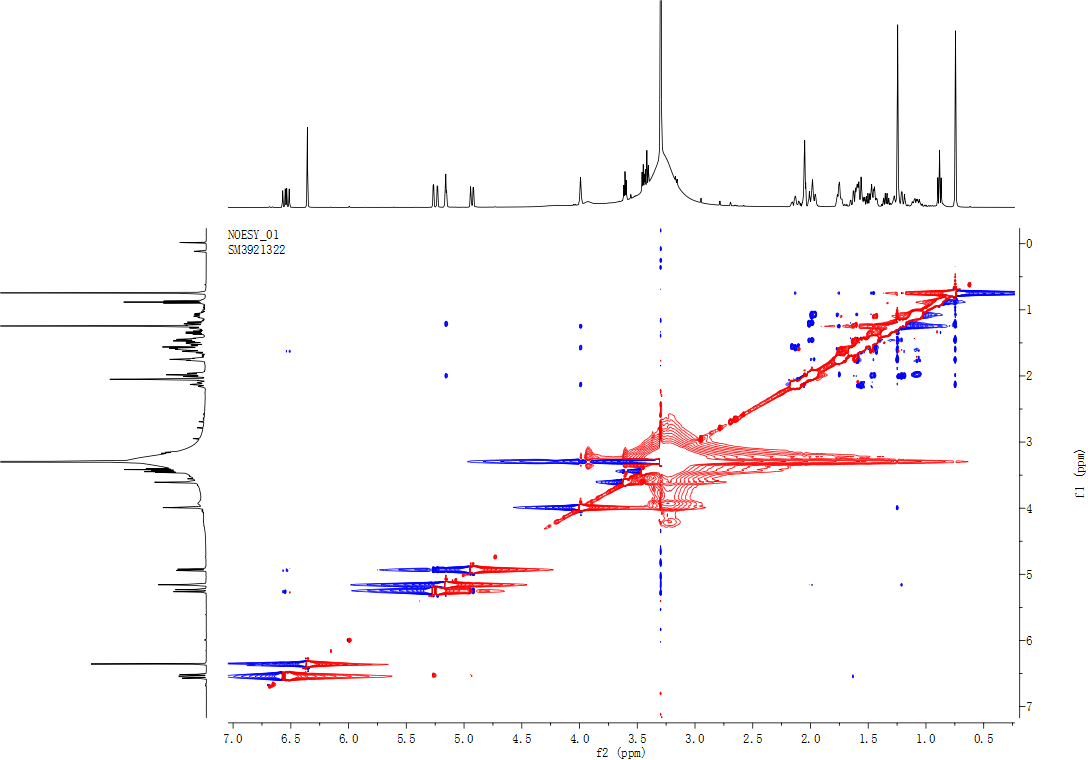


**Figure S12.** NOESY (acetone-*d*_6_) spectrum of compound **1**

**Figure S13.** ESIMS spectrum of compound **1**

**Figure S14.** HRESIMS spectrum of compound **1**

**
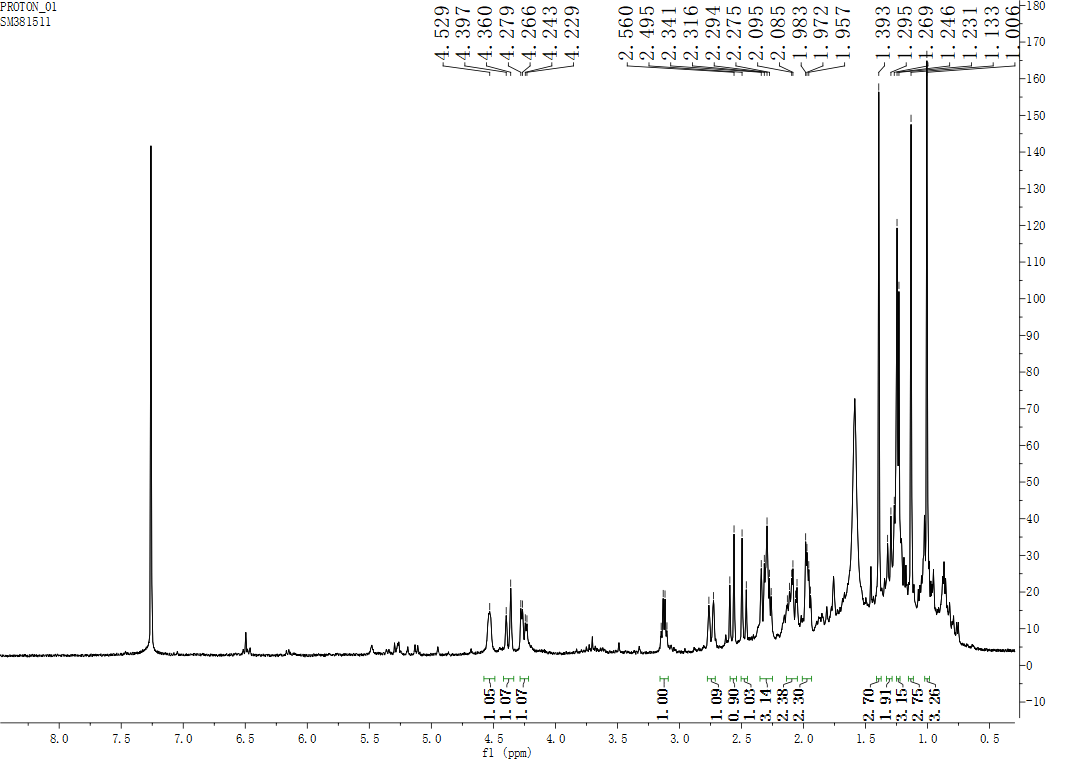
**

**Figure S15.** ^1^H NMR (500 MHz, CDCl_3_) spectrum of compound **2**


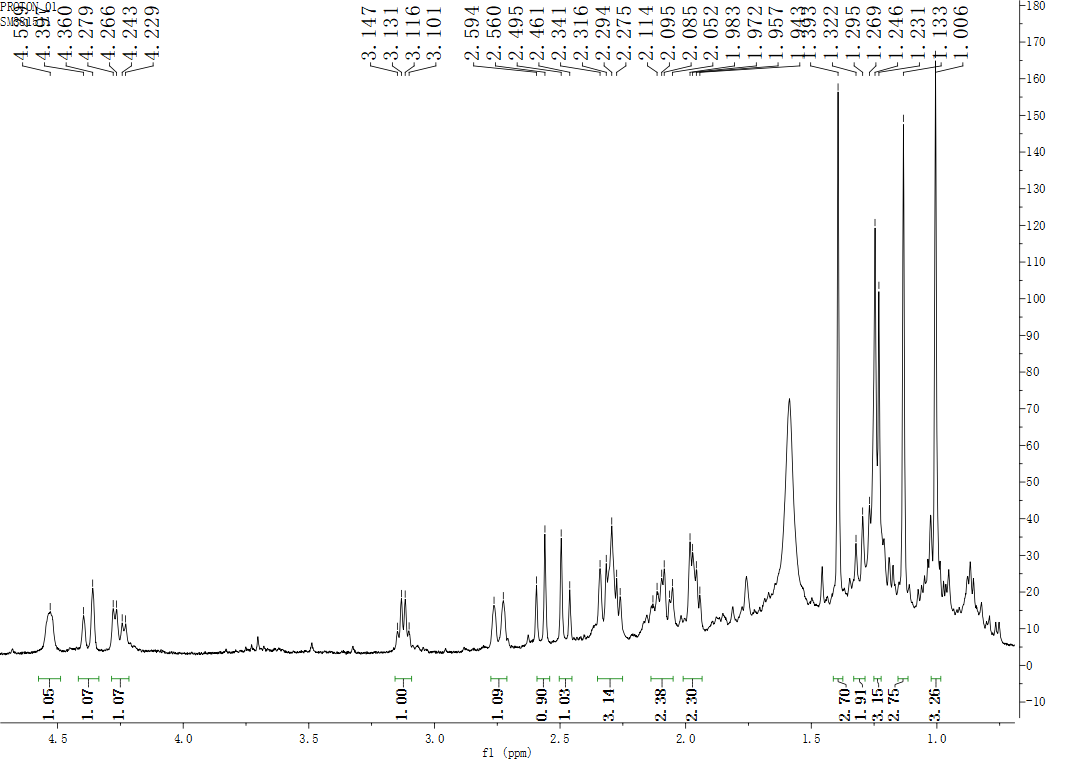


**Figure S16.** Partial ^1^H NMR (500 MHz, CDCl_3_) spectrum of compound **2**


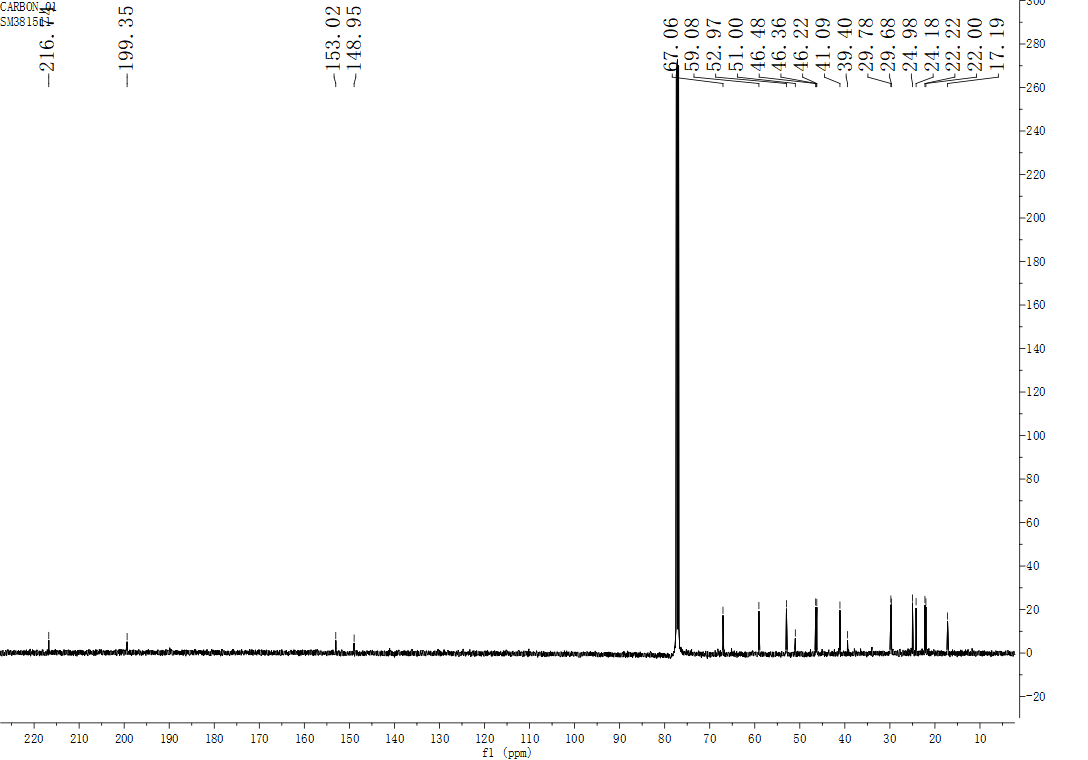


**Figure S17.** ^13^C NMR (125 MHz, CDCl_3_) spectrum of compound **2**


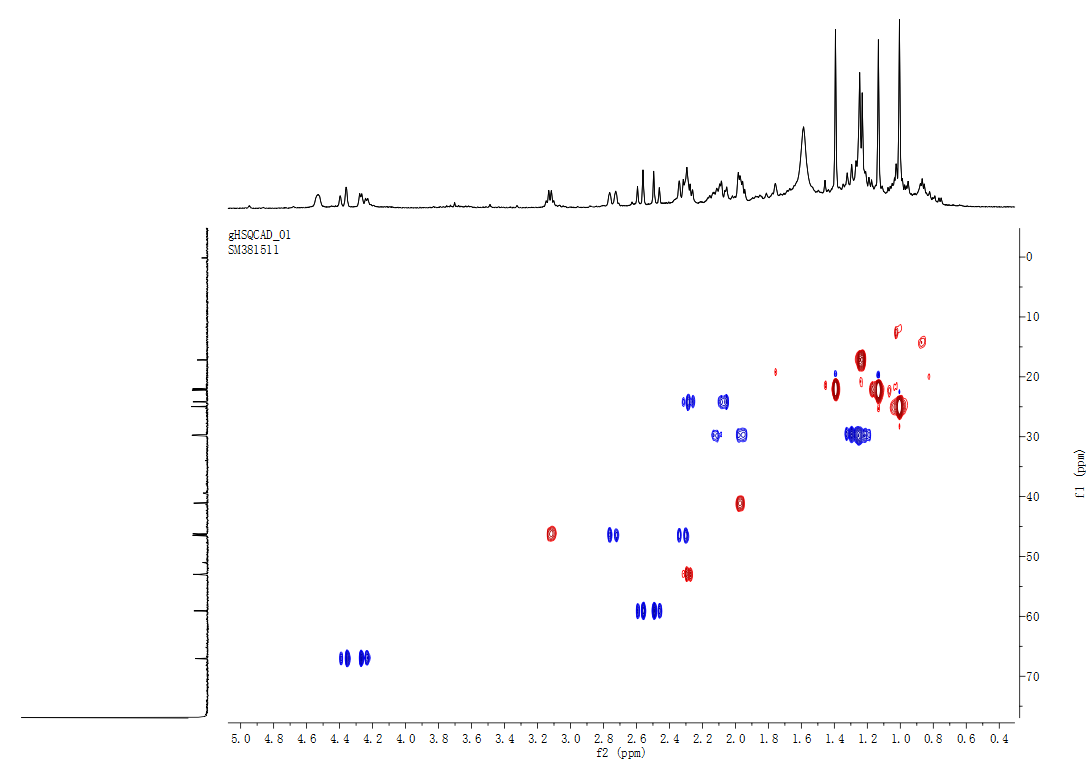


**Figure S18.** HSQC (CDCl_3_) spectrum of compound **2**


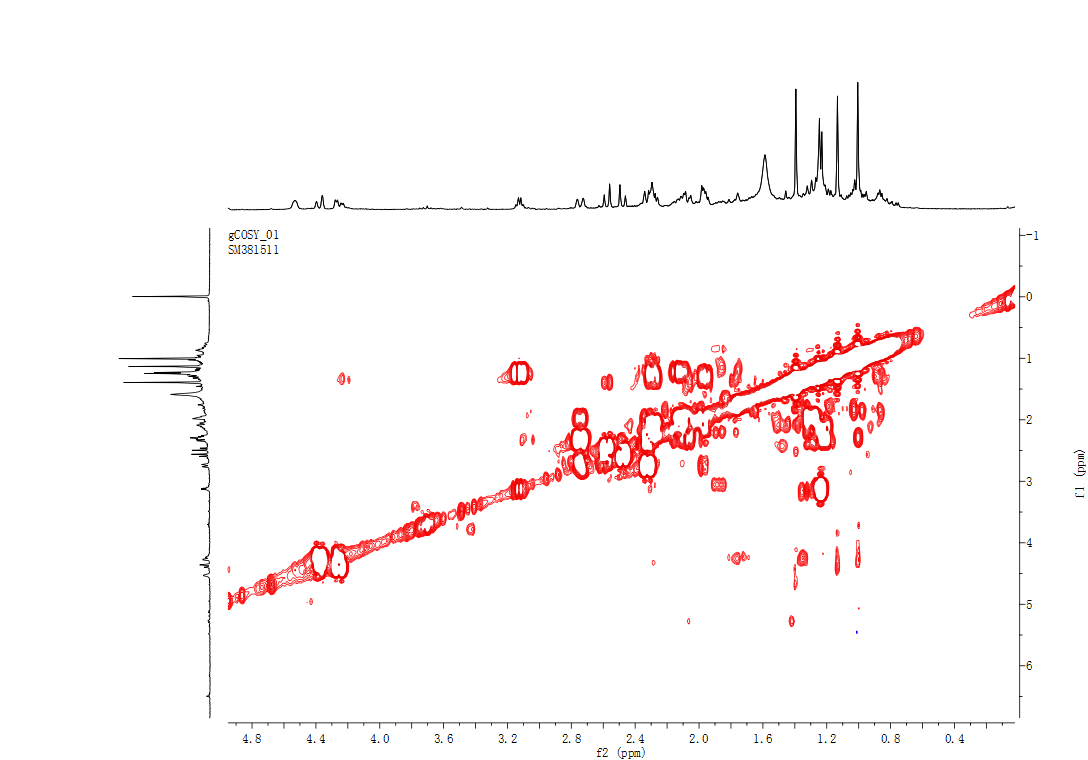


**Figure S19.** COSY (CDCl_3_) spectrum of compound **2**


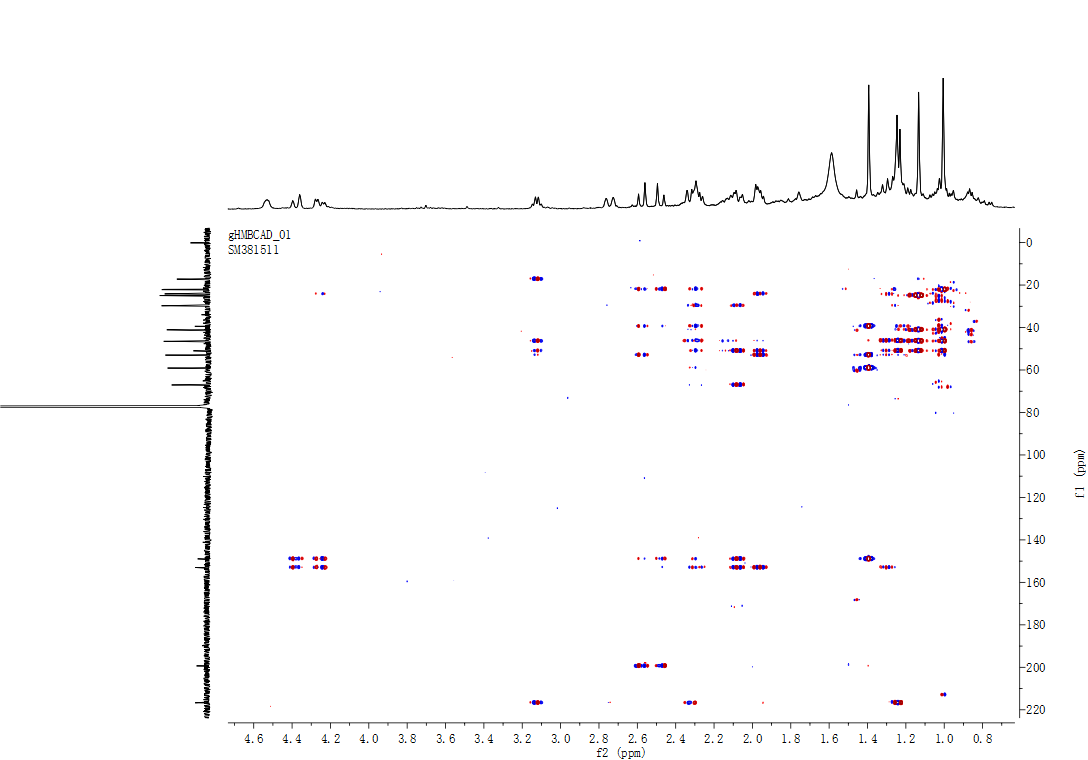


**Figure S20.** HMBC (CDCl_3_) spectrum of compound **2**


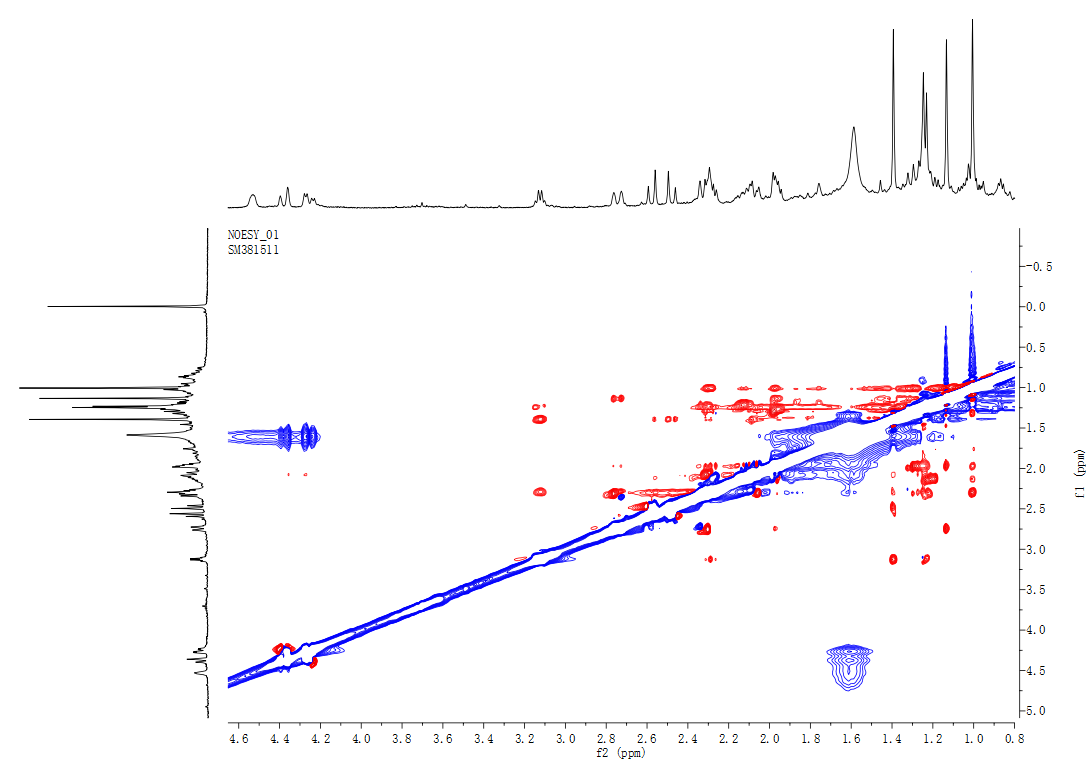


**Figure S21.** NOESY (CDCl_3_) spectrum of compound **2**


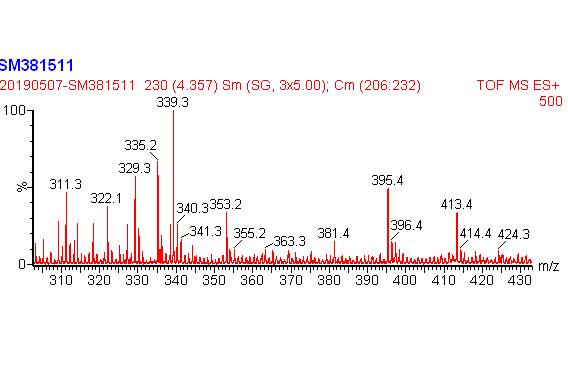


**Figure S22.** ESIMS spectrum of compound **2**

**Figure S23.** HRESIMS spectrum of compound **2**

**
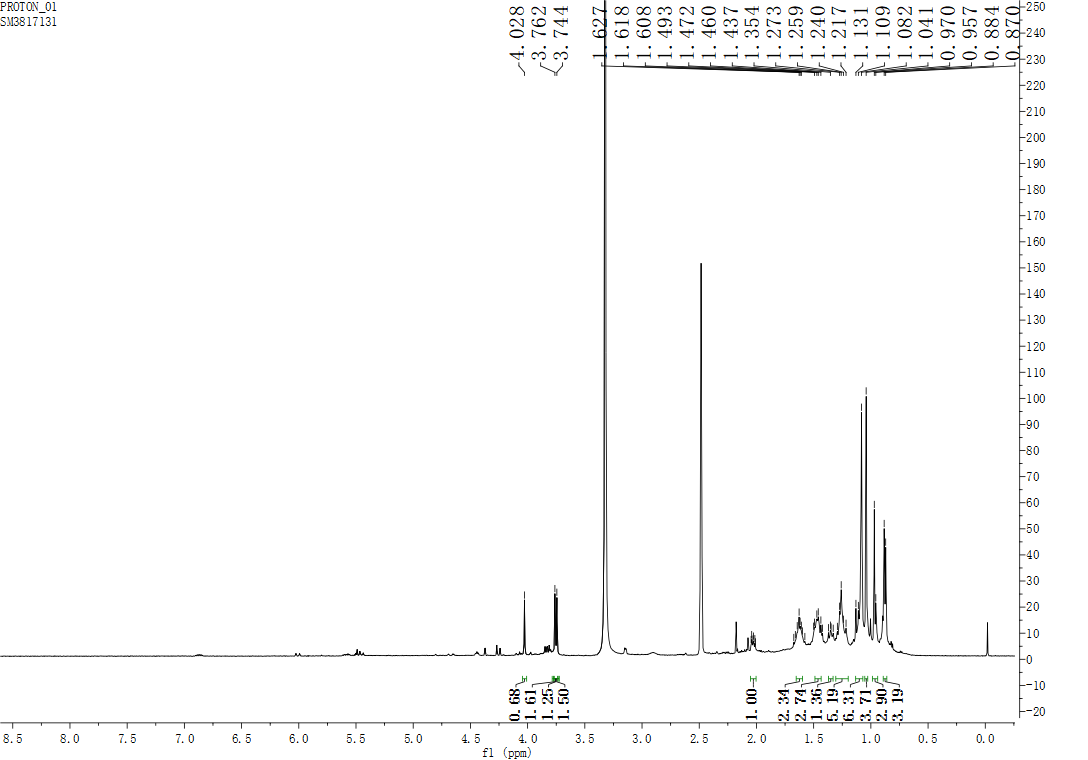
**

**Figure S24.** ^1^H NMR (500 MHz, DMSO-*d*_6_) spectrum of compound **3**


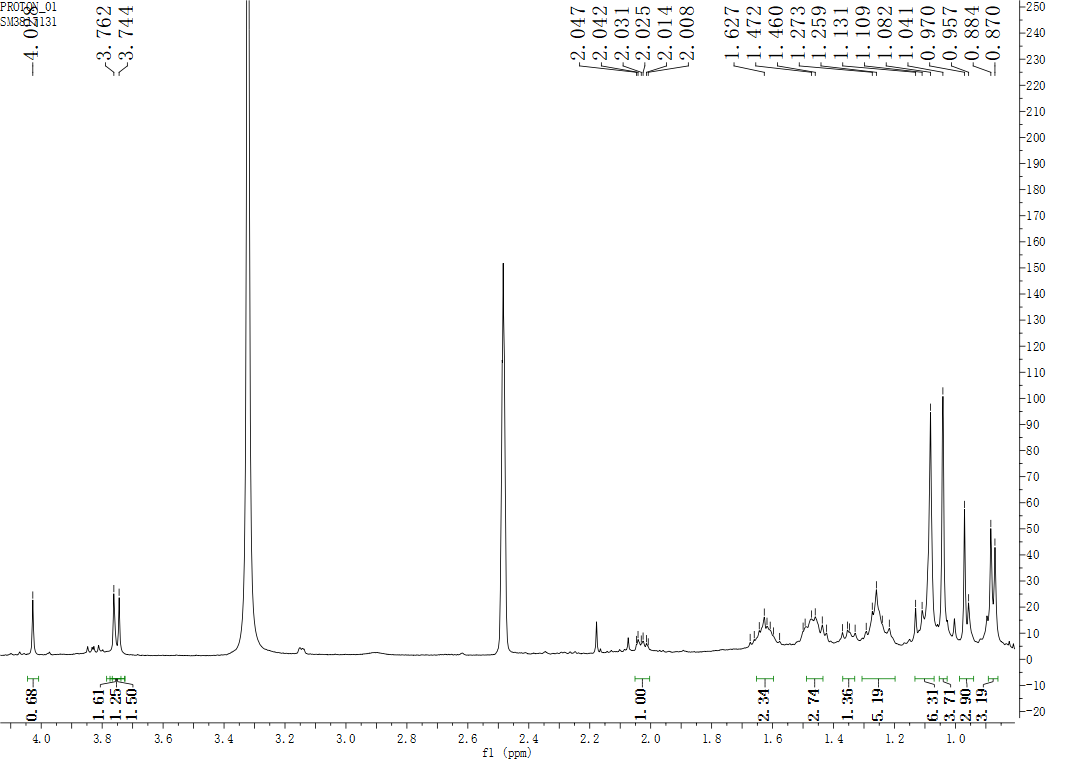


**Figure S25.** Partial ^1^H NMR (500 MHz, DMSO-*d*_6_) spectrum of compound **3**


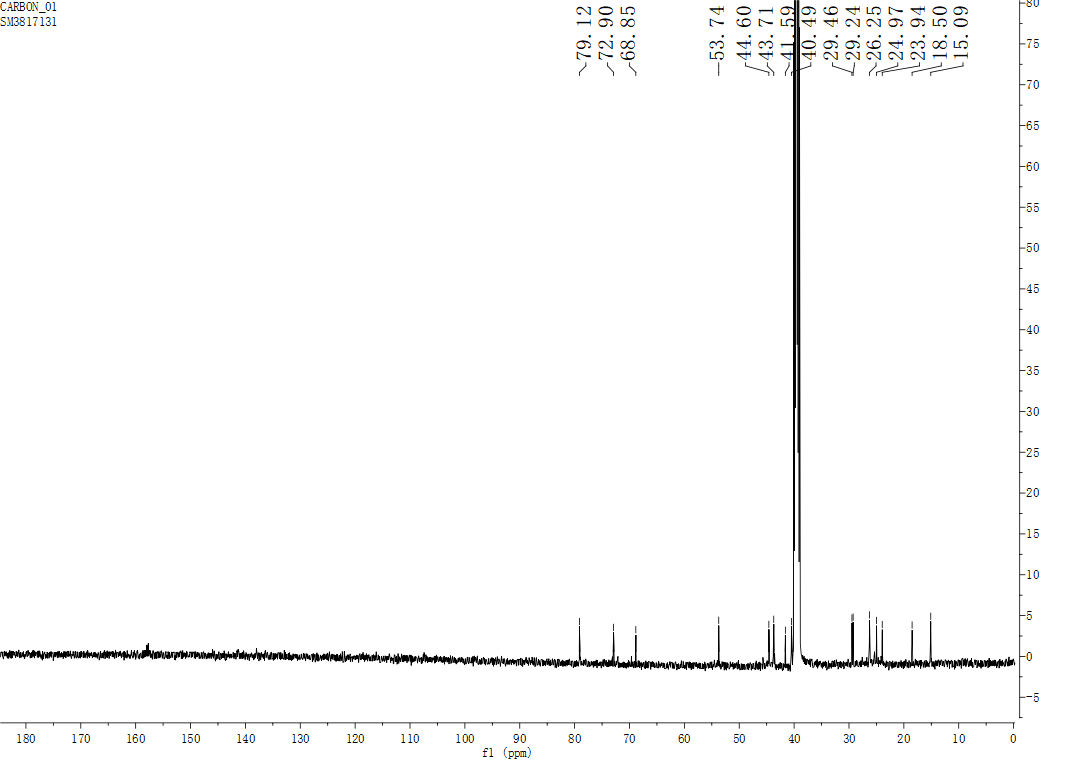


**Figure S26.** ^13^C NMR (125 MHz, DMSO-*d*_6_) spectrum of compound **3**


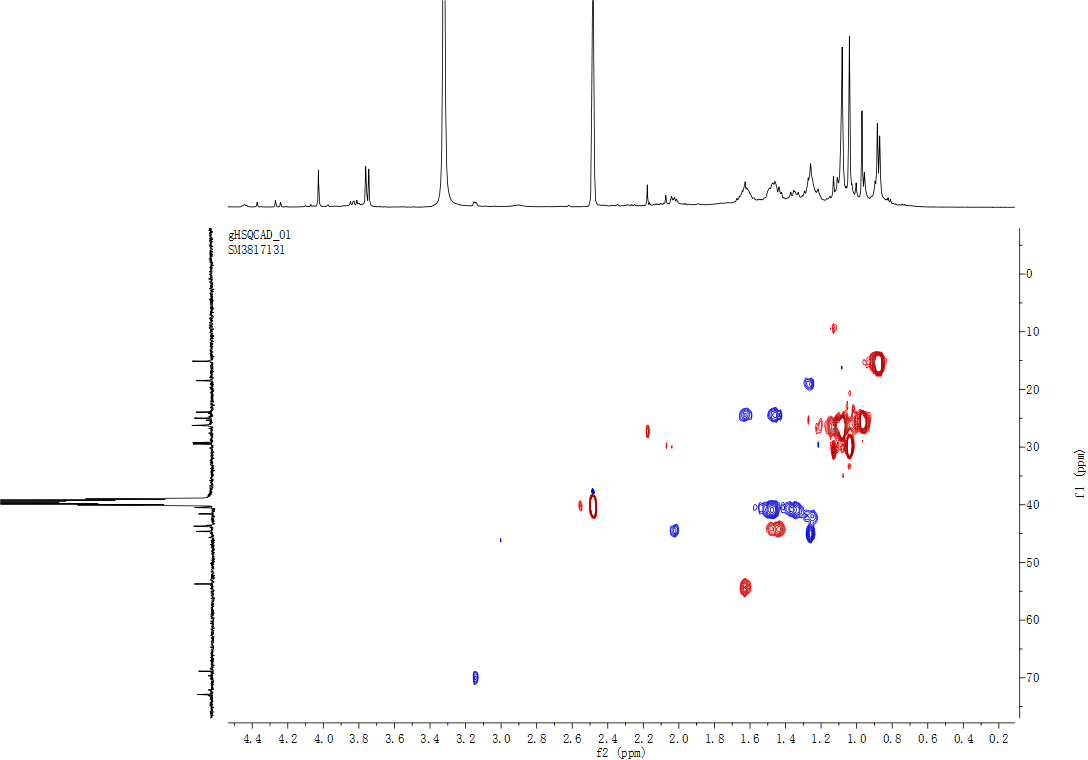


**Figure S27.** HSQC (DMSO-*d*_6_) spectrum of compound **3**


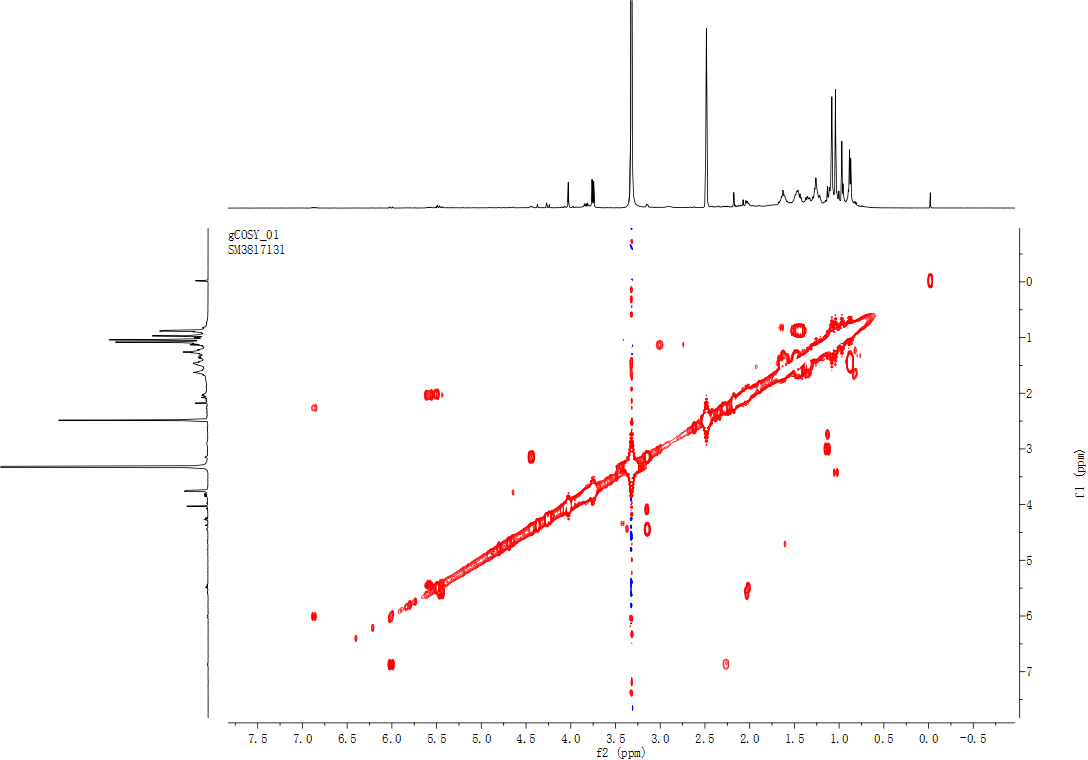


**Figure S28.** COSY (DMSO-*d*_6_) spectrum of compound **3**


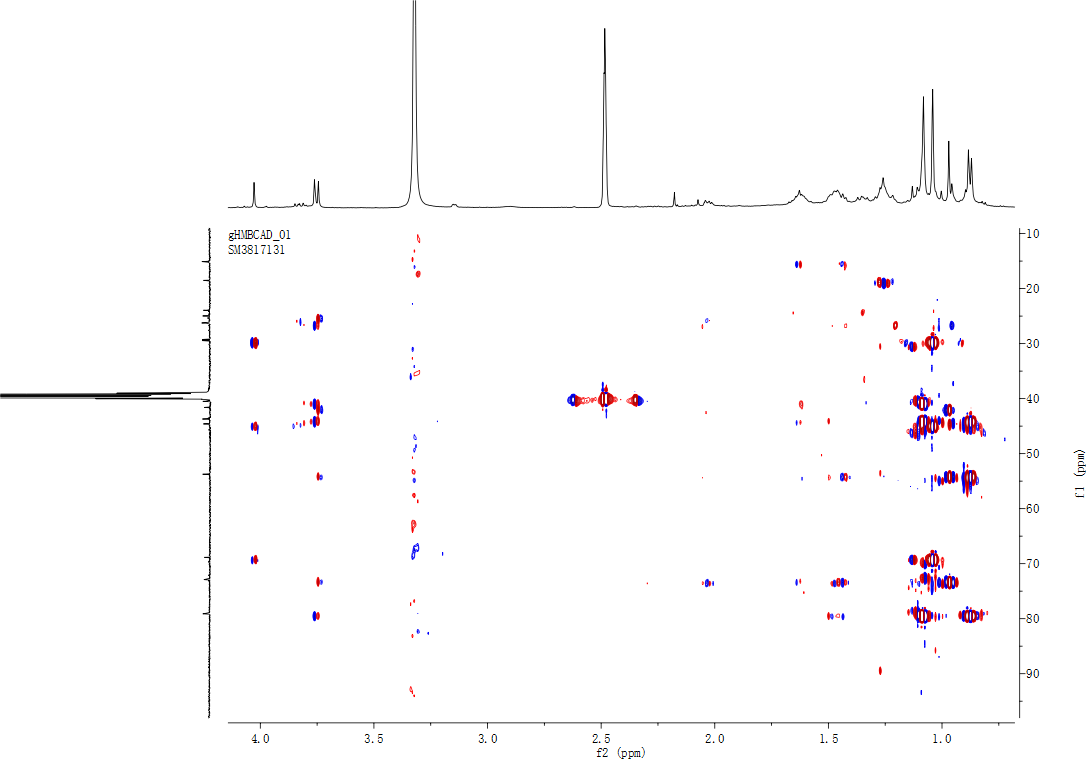


**Figure S29.** HMBC (DMSO-*d*_6_) spectrum of compound **3**


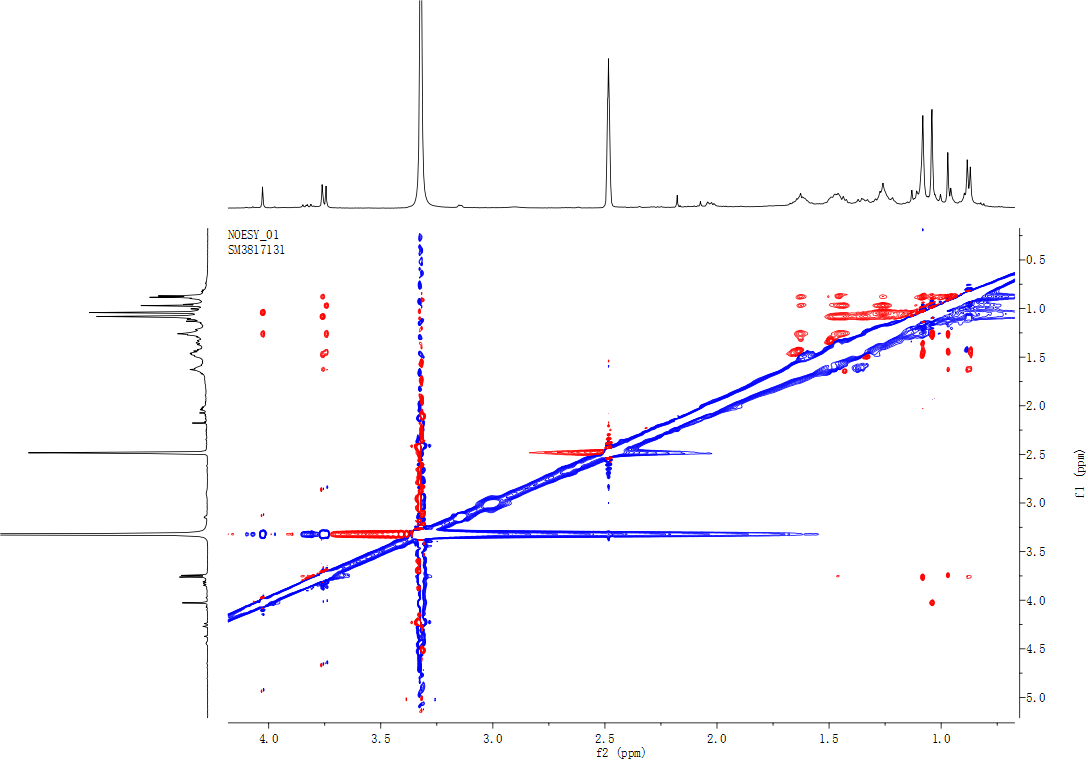


**Figure S30.** NOESY (DMSO-*d*_6_) spectrum of compound **3**


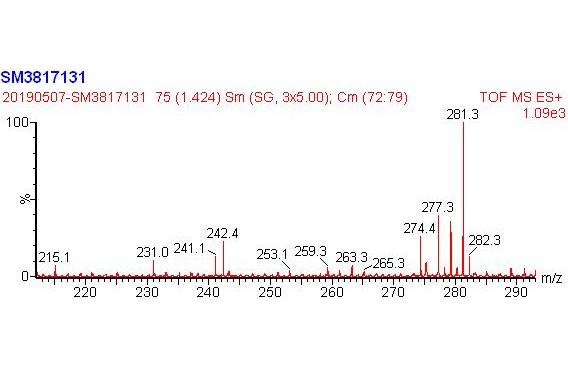


**Figure S31.** ESIMS spectrum of compound **3**

**Figure S32.** HRESIMS spectrum of compound **3**
